# A CD25-biased interleukin-2 for autoimmune therapy engineered via a semi-synthetic organism

**DOI:** 10.1038/s43856-024-00485-z

**Published:** 2024-03-26

**Authors:** Jerod L. Ptacin, Lina Ma, Carolina E. Caffaro, Nicole V. Acuff, Kristine Germar, Peter Severy, Yanyan Qu, Jose-Luis Vela, Xinming Cai, Kristine M. San Jose, Hans R. Aerni, David B. Chen, Ean Esche, Taylor K. Ismaili, Rob Herman, Yelena Pavlova, Michael J. Pena, Jasmine Nguyen, Lilia K. Koriazova, Laura K. Shawver, Ingrid B. Joseph, Jill Mooney, Mark Peakman, Marcos E. Milla

**Affiliations:** 1 Synthorx, a Sanofi Company, 11099 N. Torrey Pines Rd. Suite 190, La Jolla, CA 92037 USA; 2 Sanofi, 350 Water St., Cambridge, MA 02141 USA

**Keywords:** Drug development, Autoimmune diseases

## Abstract

**Background:**

Natural cytokines are poorly suited as therapeutics for systemic administration due to suboptimal pharmacological and pharmacokinetic (PK) properties. Recombinant human interleukin-2 (rhIL-2) has shown promise for treatment of autoimmune (AI) disorders yet exhibits short systemic half-life and opposing immune responses that negate an appropriate therapeutic index.

**Methods:**

A semi-synthetic microbial technology platform was used to engineer a site-specifically pegylated form of rhIL-2 with enhanced PK, specificity for induction of immune-suppressive regulatory CD4 + T cells (Tregs), and reduced stimulation of off-target effector T and NK cells. A library of rhIL-2 molecules was constructed with single site-specific, biorthogonal chemistry-compatible non-canonical amino acids installed near the interface where IL-2 engages its cognate receptor βγ (IL-2Rβγ) signaling complex. Biorthogonal site-specific pegylation and functional screening identified variants that retained engagement of the IL-2Rα chain with attenuated potency at the IL-2Rβγ complex.

**Results:**

Phenotypic screening in mouse identifies SAR444336 (SAR’336; formerly known as THOR-809), rhIL-2 pegylated at H16, as a potential development candidate that specifically expands peripheral CD4+ Tregs with upregulation of markers that correlate with their suppressive function including FoxP3, ICOS and Helios, yet minimally expands CD8 + T or NK cells. In non-human primate, administration of SAR’336 also induces dose-dependent expansion of Tregs and upregulated suppressive markers without significant expansion of CD8 + T or NK cells. SAR’336 administration reduces inflammation in a delayed-type hypersensitivity mouse model, potently suppressing CD4+ and CD8 + T cell proliferation.

**Conclusion:**

SAR’336 is a specific Treg activator, supporting its further development for the treatment of AI diseases.

## Plain Language Summary

Interleukin-2 (IL-2) is a protein that functions as a master regulator of immune responses. A key function of IL-2 is the stimulation of immune-regulatory cells that suppress autoimmune disease, which occurs when the body’s immune system mistakenly attacks healthy tissues. However, therapeutic use of IL-2 is limited by its short duration of action and incomplete selectivity for immune-suppressive cells over off-target immune-stimulatory cells. We employ a platform that we have previously developed, which is a bacterial organism with an expanded DNA code, to identify a new version of IL-2, SAR444336 (SAR’336), with an extended duration of activity and increased selectivity for immune-suppressive cells. In mice and monkeys, SAR’336 was a specific activator of immune suppression, with minimal effect on immune cells that stimulate autoimmunity. Our results support further development of SAR’336 for treatment of autoimmune disorders.

## Abstract (web summary)

Ptacin et al. utilize a semi-synthetic microbial platform with an expanded genetic code to discover a PEGylated IL-2 compound that stimulates Tregs, with minimal effects on effector populations. Studies in mice and primates demonstrate Treg stimulation and support development of this compound for the potential treatment of autoimmune diseases.

## Introduction

Immune homeostasis requires a delicate balance between pro-inflammatory and suppressive immune responses, and imbalances often result in immunologic disease. Cluster of differentiation (CD)4+ regulatory T cells (Tregs) play a key role in maintaining immune homeostasis by restricting the activity of self-reactive CD4+ and CD8+ effector T cells, with their disfunction associated with multiple autoimmune (AI) and inflammatory-related conditions^1–7^. The discovery and development of therapeutic agents promoting Treg cell activation and expansion to down-modulate AI responses is an investigational strategy aimed at resetting immune tolerance^2,5,8–10^.

A current focus for pharmacological Treg modulation includes cytokine therapies such as recombinant human IL-2 (rhIL-2; aldesleukin). IL-2 plays central roles in immune homeostasis via stimulation of effector CD8 + T, helper CD4 + T, and natural killer (NK) cells, and of immune suppressive Tregs. The dual functions of IL-2 present a challenge in utilizing this cytokine for the treatment of AI diseases, as IL-2 therapy can elicit distinct, opposing immune responses based upon its exposure and the differential expression of IL-2 receptor subunits on distinct cell types^2,5,8–10^.

The heterodimeric IL-2R beta and gamma chain (IL-2Rβγ) signaling complex is expressed on all T and NK cells^2–5^. Certain cell types, including Tregs, express an additional IL-2 receptor subunit, IL-2R alpha (IL-2Rα or CD25), which does not itself possess signaling function, but enhances capture of IL-2 and presentation to the signaling complex^3,5,11–14^. High constitutive expression of the IL-2Rα subunit on CD4+ Tregs allows for preferential Treg stimulation at low IL-2 levels not sufficient to significantly activate the IL-2Rβγ complex on conventional T cell or NK cell populations^3,4,8,12,15–17^.

Controlling the differential pharmacology of IL-2 is an ongoing strategy for therapeutic intervention in AI diseases. In an attempt to leverage the high potency of IL-2 for stimulation of Tregs versus effector populations, clinical development of low-dose IL-2 treatment for diverse T cell-mediated AI disorders has been explored, demonstrating therapeutic benefit^1,18–22^. While these findings indicate the promise of rhIL-2 therapy for immunologic diseases, the short half-life and dose-limiting adverse effects of rhIL-2 administration complicate treatment and limit the efficacy of this potential therapy^2,5,9^. Diverse strategies have been explored to develop variants of human IL-2 with improved pharmacological attributes and specificity for Treg cells, and significant progress has been made in this regard in recent years^9,10,23–37^. Yet, such approaches continue to remain challenging due to a paucity of methods for precisely engineering systemic half-life and target cell specificity in proteins.

We applied a microbial platform with a semi-synthetic, expanded DNA code^38^ to enable site-specific genetic introduction of click-chemistry compatible non-canonical amino acids (nAAs) into the IL-2 molecule^39^. Using this method, we generated a library of site-specific IL-2 variants covalently conjugated to polyethylene glycol (PEG), exhibiting distinct pharmacological properties. We performed an in vitro cell-based screen of IL-2 variants conjugated to PEG and identified an IL-2 variant pegylated at residue H16, peripheral to the interface with IL-2Rβ chain. In vitro binding studies showed that this compound, termed SAR’336 (formerly known as THOR-809), engaged the IL-2Rα chain without detectable affinity for IL-2Rβ. In mice and cynomolgus monkeys, administration of SAR’336 induced higher frequencies of peripheral Tregs and demonstrated an extended half-life leading to sustained peripheral exposure. In those animals, elevated Treg cell frequencies were found in the circulation, lymph nodes, spleen, and thymus, with upregulation of markers that correlate with Treg suppressive function. Tregs isolated from SAR’336 treated mice showed increased suppressive capacity over CD4+ and CD8 + T cells ex vivo. Finally, SAR’336 limited delayed-type hypersensitivity (DTH) reactions in a mouse model in vivo. Together, these findings demonstrate that SAR’336 is a specific activator of Treg cell suppressive function, with minimal change in conventional T and NK cell populations, providing rationale for the development of SAR’336 as a potential treatment for AI disorders.

## Methods

### Human donors

Human blood samples for studies reported herein were collected from de-identified healthy donors and were approved by the Institutional Review Board of The Scripps Research Institute (#17-7065). All donors provided informed consent. The studies were performed following the guidelines of the World Medical Association’s Declaration of Helsinki.

### Animal models

All procedures performed in studies involving animals reported herein were conducted following an approved Institutional Animal Care and Use Committee (IACUC) protocol, and in accordance with the ethical standards of the institution or practice at which the studies were conducted.

C57BL/6 mice were housed at 72 ± 5 °F, ambient humidity, and 12 h light:dark cycle. Animal welfare for this study complied with the U.S. Department of Agriculture’s Animal Welfare Act (9 CFR Parts 1, 2, and 3) as applicable. All experimental data management and reporting procedures were in strict accordance with applicable HD Biosciences Guidelines and Standard Operating Procedures.

Purpose-bred cynomolgus monkeys (*Macaca fascicularis*) were sourced from licensed vendors and underwent standard quarantine periods prior to study initiation. Animal studies were conducted in Association for Assessment and Accreditation of Laboratory Animal Care (AAALAC)-accredited facilities at Charles River Laboratories (Reno, NV) under protocols approved by the IACUC. 2- to 4-year-old male cynomolgus monkeys weighing 2–3 kg were used in these studies.

### Molecular biology

Primers for polymerase chain reaction (PCR) amplification were purchased from Integrated DNA Technologies (Coralville Iowa, USA). All oligonucleotides containing unnatural DNA base pairs were synthesized by Biosearch Technologies (Petaluma, California, USA) with purification by reverse phase cartridge. Phosphoramidites of dNaM and dTPT3 were synthesized by WuXi AppTec (Tianjin, China). The dNaM, dTPT3, NaM, and TPT3 nucleosides were synthesized by WuXi AppTec and triphosphates were added by MyChem LLC (San Diego, California, USA). Plasmid preparations for cloning plasmids were performed using QIAprep commercial kits (QIAGEN). All cloning enzymes were purchased from New England Biolabs (Ipswitch, Massachusetts, USA). gBlock^®^ gene fragments were purchased from IDT (Coralville, Iowa, USA). Plasmids isolation was carried out using Qiagen miniprep kits (QIAprep, Qiagen or ZR Plasmid Miniprep Classic, Zymo Research). PCR reactions were performed in TempAssure PCR 8-Tube Strips (USA Scientific, Cat. No. 1402-2380) with a Roche Life Science LightCycler 96 Q-PCR thermocycler. Zymo Research DNA Clean and Concentrator kits were used for purification of Golden Gate Assembly reactions, vector and insert fragments. Gibson and Golden Gate Assembly protocols were performed on an MJ Research PTC-200 Thermal Cycler.

Inserts for Gibson Assembly of the IL-2 genes as well as the anticodon of the *M. mazei pylT* gene were produced by PCR amplification of chemically synthesized primers using dTPT3TP and dNaMTP. Primers contained BsaI sites to produce distinct overhangs which complement the cloning region of the destination plasmid. Insert template oligos were amplified at 1 ng per 50 µL reaction using a mixture of OneTaq DNA Polymerase and Deep Vent Polymerase (NEB), 0.5 µM of each primer, 1x OneTaq buffer, 0.5x of SybrGreen, 3.0 mM MgSO4, 200 µM dNTPs, 100 µM of each unnatural nucleotide. Inserts were purified using the Zymo Clean and Concentrator Kit.

Golden Gate Assembly of the IL-2 expression constructs was performed using 300 ng of vector plasmid with 25 ng of both *M. mazei pylT* gene and modified IL-2 coding region inserts. The IL-2 entry vectors were linearized by PCR amplification using 0.02 U/µL Q5 DNA Polymerase with 200 µM dNTPs, 1× Q5 Reaction buffer, 0.5× SYBR Green, 2 ng of template per 50 µL reaction and 0.5 µM of primers which amplify from the IL-2 Golden Gate entry site. The resulting product was purified using ZymoClean PCR purification kit. Vector DNA (300 ng) was combined with 25 ng of pylT fragment and IL-2 insert, 0.67 U/µL T4 DNA ligase, 0.67 U/µL BsaI-HF, 1x CutSmart buffer and 1 mM ATP in 30 µL. Following the Golden Gate reaction, the mixture was incubated with T5 exonuclease and KpnI at 37 °C for one hour. Finally, the in vitro assembled plasmid was purified using a ZymoClean PCR purification kit.

### Generation of expression strains

To generate the azide-containing nAA, N6- (2-azidoethoxy)-carbonyl-L-lysine (AzK)-substituted IL-2 expression strains, an overnight culture of the parental strain SYTX169 was inoculated into 2xYT media containing 5 µg/mL chloramphenicol, 5 µg/mL tetracycline, and 50 mM potassium phosphate. The next day the culture was diluted and grown until an optical density of 0.4. The cells were then chilled on ice and centrifuged at 3000 × *g* for 10 min at 4 °C. The resulting cell pellet was washed twice using an equal volume of pre-chilled sterile deionized water and centrifuged. After the second wash, the cells were resuspended in fresh pre-chilled sterile deionized water and transformed via electroporation. After electroporation, pre-warmed 2xYTP with 5 µg/mL chloramphenicol was added to the electroporated cells and rescued at a final concentration of 150 µM dNaMTP and 37.5 µM dTPT3TP at 37 °C shaking at 250 rpm for an hour and inoculated into 2xYTP containing 5 µg/mL tetracycline, 5 µg/mL chloramphenicol, 50 µg/mL zeocin, 150 µM dNaMTP, and 37.5 µM dTPT3TP.

### Preparation of pegylated IL-2 variants

Expression of IL-2 variants with AzK substitutions was performed in 2xYT medium (Thermo Fisher Scientific, Cat. No. BP9736) supplemented with 50 mM potassium phosphate, 100 µg/mL ampicillin, 5 µg/mL chloramphenicol, 50 µg/mL Zeocin, 150 µM dNaMTP, and 37.5 µM dTPT3TP. Expression cultures were incubated overnight at 37 °C, and diluted prior to reaching OD600nm of 1 before dilution in the same medium back to OD600nm of 0.05. Upon reaching OD600nm of ~0.8, cultures were pre-induced with 250 µM NaMTP, 25 µM TPT3TP, and 10 mM AzK-HCl prepared in deionized water (Synchem, Buffalo Grove, IL, USA; Cat No. 36462). Approximately 15 min after pre-induction, cultures were induced with 1 mM IPTG and incubated for an additional 5 h. Cultures were collected by centrifugation at 6000 rpm for 20 min at 4 °C, and pellets were stored at −80 °C until use.

Inclusion bodies were generated by addition of 50 mL lysis buffer (1x PBS, Thermo Fisher Scientific, Cat. No. BP2940-4) containing protease inhibitors (Thermo Fisher Scientific, Cat. No. A32965) and 1x lysozyme (Thermo Fisher Scientific, Cat. No. 89833). Resuspended pellets were lysed via microfluidizer (Dyhydrodymatics, model M110L). Lysed samples were centrifuged at 30,000 × g for 25 min at 4 °C before discarding the supernatant. Inclusion body pellets were solubilized by addition of 25 mL 6 M Guanidine-HCl in 100 mM Tris-HCl pH8, 20 mM imidazole per liter, pipetting until homogeneity before agitation for 15 min on a rotator. Samples were centrifuged at 30,000 × *g* for 20 min at 4 °C, and pellets were discarded.

Ni-NTA resin (Thermo Fisher Scientific, Cat. No. 25216) was pre-washed with solubilization/wash buffer (6 M Guanidine HCl, 100 mM Tris-HCl pH 8, 20 mM imidazole). Equilibrated resin was added to solubilized sample in conical tubes and incubated 1 h at 4 °C on a rotator. Resin was collected by centrifugation for 2 min at 250 × *g*, and washed with 10-15 column volumes of solubilization/wash buffer, transferred to a collection column and washed for an additional 10 column volumes of solubilization/wash buffer. The column was eluted using 3 column volumes of elution buffer (1× solubilization/wash buffer containing 500 mM imidazole). Eluates were dialyzed overnight in 12 L of 20 mM Tris-HCl pH 8/150 mM NaCl, then moved to12 L of 20 mM Tris-HCl pH 8/50 mM NaCl for an additional 4 h. The resulting dialysate was centrifuged at 30,000 × *g* 20 min at 4 °C and supernatant was collected.

Samples were supplemented with 0.5 µl enterokinase (New England Biolabs, Ipswich, MA, Cat. No. P8070L) per 10 µg of protein and incubated overnight at room temperature. To initiate pegylation, 5 mM DBCO-mPEG (Click Chemistry Tools, Scottsdale, AZ; 30 kDa: Cat. No. A121, 10 kDa: Cat. No. A119, or 5 kDa: Cat. No. A118) stock was added to a final concentration of 50 µM, and incubated overnight at 4 °C. Samples were concentrated using an Amicon-15 before loading onto HiLoad 16/600 Superdex 200 pg size-exclusion chromatography column (GE Healthcare) using an ÄKTA™ Pure system (GE Healthcare) in 1x PBS. Fractions were analyzed by SDS-PAGE and Western Blot using rabbit anti-IL-2 oligoclonal antibody (Thermo Scientific, Cat. No. 710146). Peak fractions were pooled and adjusted to a final concentration of 4.5% acetonitrile, 0.043% trifluoroacetic acid. Samples were loaded onto a reversed phase chromatography column (GE Healthcare) in 4.5% acetonitrile/0.043% trifluoroacetic acid and eluted using a gradient of buffer B (90% acetonitrile/0.028% trifluoroacetic acid). Fractions of interest were pooled and mixed 1:1 with deionized H20, and lyophilized using a Labconco FreeZone 4.5. Lyophilized samples were then resuspended in 50% acetonitrile 0.1% TFA and quantitated using a bovine serum albumin (BSA) standard curve using bicinchonic acid (BCA) method to determine final concentration prior to final lyophilization as above. Samples were stored lyophilized at −80 °C until use. Preparation of non-pegylated IL-2 and muteins was performed as above without pegylation steps described.

### Surface plasmon resonance (SPR)

The SPR studies described herein were performed under contract by Biosensor Tools LLC (Salt Lake City, Utah, USA). The molecular mass of IL-2 samples was assumed to be 15.5 kDa, plus additional size of 30 kDa or 50 kDa PEG conjugation. 50-µg samples were dissolved in 50 µL water to make stock solutions of 64.5 µM. The Fc-tagged human IL-2 receptor subunits (extracellular domains) were purchased from Sino Biological (hIL-2 Rα-Fc Sino 10165-H02H, hIL-2 Rβ-Fc Sino 10696-H02H). Recombinant human IL-2 (hIL-2) was purchased from Thermo Scientific (Cat. No. PHC0021) and prepared at a stock concentration of 64.5 µM.

Using a Biacore 4000 optical biosensor, the Fc-IL-2 receptor subunits were immobilized to a protein A-coated C1 sensor chip to densities of ~300–800 RU and equilibrated with running buffer (10 mM HEPES, 150 mM NaCl, 0.005% Tween-20, 0.1 mg/mL BSA, pH 7.4). For additional higher density surface studies for human and cynomolgus β receptors, the receptors were captured to ~8000 and ~13,000 RU, respectively. Binding studies were performed at 25 °C. When necessary, the protein A surfaces were regenerated with 150 mM phosphoric acid between binding cycles. The test articles were tested in duplicate, in twofold dilution series from 0.7 to 200 nM. The responses from IL-2Rβ surfaces were fit to a 1:1 interaction model to obtain binding parameters using Scrubber (v2.0c).

### Flow cytometry assay for pSTAT5 induction

Primary human peripheral blood mononuclear cell (PBMC) studies using flow cytometry were performed under contract by PrimityBio (Fremont, California, USA) or internally. The collection of blood samples from healthy donors was approved by the Institutional Review Board of The Scripps Research Institute (#177065). Leukocyte reduction systems (LRS) were purchased from Cell IDX (San Diego, California, USA). All donors provided informed consent. The studies were performed following the guidelines of the World Medical Association’s Declaration of Helsinki.

Whole blood samples were treated with compound and analyzed for pSTAT5 expression as previously described^39^. Briefly, blood samples were pre-warmed to 37 °C, treated with a serial dilution of compound for 45 min, then red blood cells were lysed, and the leukocytes were stained with a panel of markers. Tregs are defined as CD3 + CD4+CD127loCD25+ and CD8 T cells are CD3+CD8+.

### Quantitative analysis of IL-2 by ELISA

Bioanalysis of plasma samples was performed using a human IL-2 ELISA assay (Abcam, Cat. No. 10056). Concentrations of SAR’336 in plasma and the internal standard were determined using the ELISA assay. Pharmacokinetic (PK) data analysis was performed at NW Solutions (Seattle, Washington, USA).

### Animal models

Purpose-bred cynomolgus monkeys (*Macaca fascicularis*) were sourced from licensed vendors and underwent standard quarantine periods prior to study initiation. Animal studies were conducted in AAALAC-accredited facilities at Charles River Laboratories (Reno, NV) under protocols approved by the Institutional Animal Care and Use Committee. Two- to four-year-old male cynomolgus monkeys weighing 2–3 kg were used.

All animals and data points were included in the analysis, except in cases of poor sample quality such as clotted blood. Sample sizes were not statistically pre-determined. Single-dose PK/pharmacodynamic (PD) studies utilized *n* = 3–4 animals per group. Repeat dosing studies or studies dosing with antigen utilized *n* = 4–10 animals per group due to larger expected variability. In total, 265 mice and 48 monkeys were used. Samples/animals in all experiments were randomized to each group. Animals were allocated to study groups at random by the vendor or assigned associate and did not control for confounders such as order of treatments. Studies included serial collections or individual animals per time point. Most studies report quantitative data measured without subjective scoring; the exception was a qualitative blinded analysis by a pathologist to score ear inflammation. Vendors, investigators, and data analysts were not blinded during experiments.

### Blood and tissue collection and processing for PK and immune cell profiling

C57BL6 mice were housed at 72 ± 5 °F, ambient humidity, and 12 h light:dark cycle. Animal welfare for this study complied with the U.S. Department of Agriculture’s Animal Welfare Act (9 CFR Parts 1, 2 and 3) as applicable. All experimental data management and reporting procedures were in strict accordance with applicable Crown Bioscience, Inc or HD Biosciences Guidelines and Standard Operating Procedures.

After a single intravenous injection of mice with H16, terminal blood samples were collected by cardiac puncture following CO_2_ euthanasia. For the study of naïve mice, blood was collected at 13 time points (0.03, 0.17, 0.5, 1, 2, 4, 8, 12, 24, 48, 72, 96, and 120 h) post-dose, sacrificing 3 mice per time point. Whole blood and plasma samples were analyzed for pSTAT5 expression across immune cell subsets and concentrations of IL-2 as previously described^39^. Alternatively, C57BL/6 mice were given a single dose of SAR’336 subcutaneously (SC) at 0.3 mg/kg. At 3-, 8-, and 14-days post-dose, whole blood was collected from groups in K2 EDTA tubes followed by necropsy and collection of spleen, thymus, and lymph node into cold phosphate buffered saline (PBS) with 5% fetal bovine serum and then disaggregation by 70-micron filter. Blood and spleen samples were pre-lysed with ammonium chloride potassium (ACK) buffer (Gibco). Blood and tissue cells were stained with antibodies against mouse CD3, CD4, CD8, CD25, CD19, NK1.1, and forkhead box protein 3 (FoxP3) cells to assess levels of Treg (CD4+, CD25+, FoxP3+), CD4, CD8, and NK cells. Antibodies used in these studies are listed in Supplementary Table 1. Representative gating strategy is shown in Supplementary Fig. 1.

### Flow cytometry of NHP whole blood samples

Blood samples were serially collected to evaluate the effects of SAR’336 on immune cells with flow cytometry. Peripheral blood samples were treated for red blood cell lysis and fixed with Phosflow™ Lyse/Fix buffer (BD Biosciences, NJ) immediately after collection. Cells were blocked with human TruStain FcX (BioLegend, San Diego, CA) before staining for cell surface markers.

The following surface markers were used to identify immune cell types: T cells (CD3+ [BD Biosciences, clone SP34-2], CD4+ helper T cell (CD3+/CD4+ [BioLegend, clone OKT4]), CD8 T cells (CD3+/CD8 [BD, clone SK1]), CD4+ Treg cells (CD3+/CD4+/CD25+ [BioLegend, clone M-A251]/FoxP3+ [BioLegend, clone 259D]), and NK cells (CD3-/CD7+ [BD, clone M-T701]). Cells were permeabilized and stained internally with anti-pSTAT5 (Y694) antibody (BD, clone 47/Stat5(pY694) and anti-Ki67 [Thermo Fisher, clone SolA15] antibodies. Samples were acquired on a BD Fortessa flow cytometer. Antibodies are listed in Supplementary Table 1. Representative gating strategy is shown in Supplementary Fig. 1.

### DTH model

C57BL/6 mice (*n* = 10 per treatment group) were sensitized SC with 250 µg keyhole limpet hemocyanin (KLH) in PBS emulsified with equal parts  of complete and incomplete Freund’s adjuvant (0.1 mL total volume) on day 1 and challenged with 10 µg KLH/10 µl PBS intradermally in ear pinnae on day 7. Ear thickness was measured prior to KLH challenge on day 7 and daily until day 10. Groups were dosed SC 10 mL/kg on day 0 and 3 with SAR’336 at 0.03, 0.1, and 0.3 mg/kg with a vehicle control (10 mM histidine, 5% sorbitol, 0.01% polysorbate 80). A group was treated with cyclosporin A on days 0–9 at 60 mg/kg by oral gavage (10 mL/kg in 0.5% methyl cellulose). Another vehicle control group received day 7 KLH challenge without day 1 sensitization. Ear thickness of the ear flap was measured in millimeters using an engineering micrometer prior to KLH challenge on day 7 and then subsequently on days 8-10. The animals were anesthetized by isoflurane inhalation.

Whole blood from half of the treatment group was serially collected in K2 EDTA tubes at baseline and on day 7 via jugular venipuncture, and blood from the other half of the treatment group was collected on days 3 and 10. Staining antibodies for cell surface markers were added to 50 µL Brilliant Violet Buffer Plus (BD cat no. 566349) to make a master staining mix. Whole blood was incubated for 30 min in the dark at room temperature. After incubation, 1X erythrolyse solution (Serotec BUF04) was added to samples, mixed, and incubated for 10 min at room temperature. Samples were washed, resuspended in cold BD Pharmingen Mouse FoxP3 Fixation Buffer (51-9006124), and incubated for 30 min in the dark at 4 °C. Samples were incubated with FoxP3 for 30 min, washed, and acquired on a BD LSR Fortessa Flow Cytometer.

On day 10, ear pinnae samples from all mice were formalin-fixed paraffin-embedded and sectioned at 5 microns for hematoxylin and eosin staining. Sections were blindly scored by a pathologist for overall severity of inflammatory infiltrates, hyperkeratosis, epidermal hyperplasia, and corneal pustules.

### Mouse CD4 T cell suppression assay

FoxP3 eGFP mice (*n* = 4 per group) were treated with vehicle or 0.3 mg/kg SAR’336 via SC injection. Two days after injection, splenocytes were harvested and pressed through a 40 μM filter. Splenocytes were enriched for T cells using an EasySep Mouse T cell enrichment kit (Stem Cell Technologies) and subsequently stained with viability dye and a cocktail of mouse antibodies for CD4, CD25, and CD45RB. Live CD4+CD45RBloCD25+FoxP3eGFP+ Tregs from all mice and live CD4 + CD25-FoxP3eGFP- conventional cells from vehicle-treated only were sorted with a Sony SH800 cell sorter. A 1:5 ratio of latex beads pre-coated with anti-mouse CD3 (145-2C11) and anti-mouse CD28 (37.51) to CD4 T cells were co-cultured with CD4 Tregs at the indicated ratios for 3 days in complete medium in a 96-well round-bottom plate. Eight hours before harvesting plates, 0.1 µCi of 3H thymidine was added to each well. Cells were harvested and analyzed using a Perkin Elmer Microbeta microplate counter. Antibodies are listed in Supplementary Table 1. Normalized percentage of proliferation is calculated using the equation (cpm of Tconv cells treated with Treg/avg cpm of Tconv cells alone)*100, where cpm is counts per minute and Tconv refers to conventional T cells.

### Mouse CD8 T cell suppression assay

Naïve C57BL/6 mice were treated with vehicle, 0.1 mg/kg or 0.3 mg/kg SAR’336 via SC injection. Two days after injection, splenocytes were harvested and pressed through a 70 µM filter. Cells were treated with 1X RBC lysis buffer (Invitrogen) for 2 min, washed in PBS, and counted. Splenocytes were stained with a cocktail of mouse antibodies for CD4, CD25, and CD127. CD4 + CD25 + CD127- Tregs were sorted with a Sony SH800 cell sorter. Twelve vehicle-treated mice and 8 SAR’336-treated mice were used to isolate enough Tregs for the assay. Splenocytes from an additional twelve naïve C57BL/6 mice were similarly processed into a single cell suspension. CD8 T cells were isolated using a CD8 T cell isolation kit (StemCell) and labeled with CellTrace Violet™ (CTV, ThermoFisher), following manufacturer’s guidelines. A 1:1 ratio of Dynabeads^®^ (ThermoFisher) to CD8 T cells was co-cultured with CD4 Tregs at the indicated ratios for 3 days in X-Vivo™ 15 medium + 10% human serum. After 3 days, cells were stained with a viability dye and anti-CD8 antibody and run on an Attune NxT flow cytometer. Samples are gated on live CD8 + T cells for loss of expression of CTV. Antibodies are listed in Supplementary Table 1.

### Human Treg suppression assay

Fresh PBMC were enriched using StemCell SepMate50 tubes, following manufacturer’s guidelines. Cells were washed, counted, and stained for 15 min with a cocktail of antibodies for CD3, CD4, CD8, CD56, CD127, and CD25 in PBS + 1 mM EDTA + 25 mM HEPES + 1% FBS. PBMC were washed twice and filtered through a 70 µM filter prior to sorting on a BD FACSAria II Cell Sorter S10 RR027366. Live cells were defined as propidium iodide negative. CD8 T cells were defined as CD3 + CD56-CD8+ and Tregs as CD3 + CD56-CD4 + CD127-CD25+. CD8 T cells were then labeled with Cell Trace Violet (ThermoFisher), following manufacturer’s guidelines. CD8 T cells and Tregs were combined at the indicated ratios and cultured in complete RPMI (RPMI + 10% FBS + 1% penicillin/streptomycin) with rhIL-2 (Gibco Cat. No. PHC0023 or Cat. No. PHC0027) or SAR’336 on tissue culture treated plates pre-coated with purified CD3 (UCHT1) and CD28 (CD28.2) antibodies. Cells were cultured for 4 days and then stained with a cocktail of antibodies to measure proliferation by flow cytometry. Samples were acquired on an Attune NxT flow cytometer and are gated on live CD8 + T cells for loss of expression of CTV. Antibodies are listed in Supplementary Table 1.

### Statistics

Statistical analysis was performed using GraphPad Prism. EC50 values were calculated using 4-parametric logistic curves. Animal studies utilized one-way ANOVA with Dunnett post-hoc test for statistical analysis. Treatment groups were compared to vehicle control. Significance was defined as *p* < 0.05.

### Reporting summary

Further information on research design is available in the Nature Portfolio Reporting Summary linked to this article.

## Results

### Identification of pegylated IL-2 compounds that are IL-2Rαβγ agonists with reduced IL-2Rβγ engagement

**Fig. 1:**
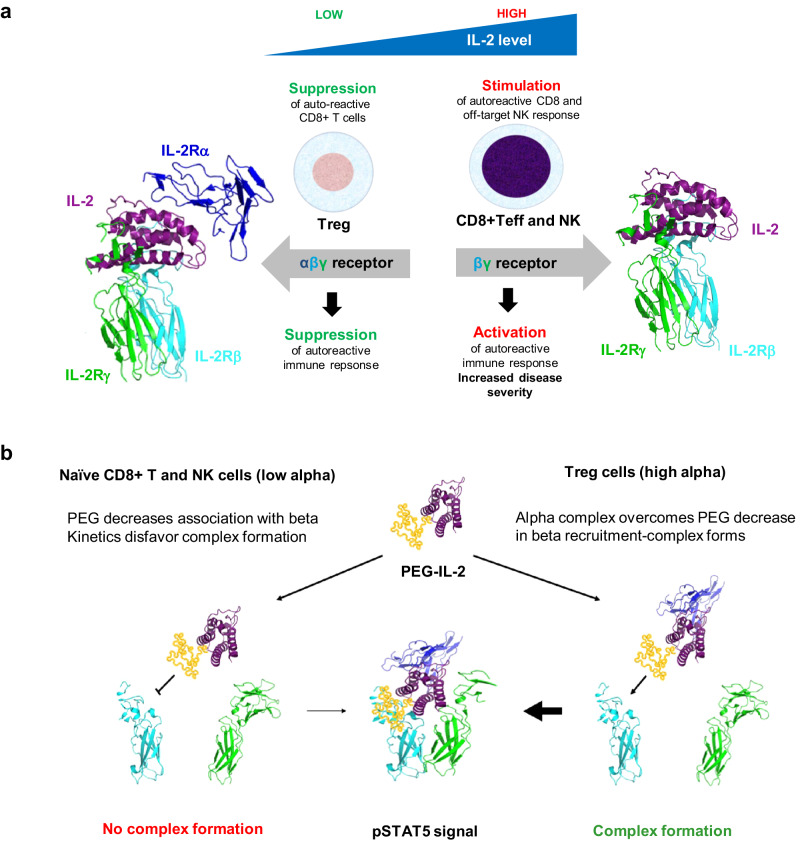
Overview of IL-2 related Treg biology and therapeutic hypothesis of an IL-2Rα biased IL-2 for Treg expansion and activation. **a** Normal Treg biology showing opposing pleiotropic effects of IL-2. **b** Schematic representation of an IL-2Rα biased IL-2 for therapeutic expansion and activation of Treg cells and induction of immune suppressive effects along with reduced stimulation of CD8 + T and NK cells. CD8 cluster of differentiation 8, IL-2 interleukin-2, IL-2Rα interleukin-2 receptor alpha chain, IL-2Rβ interleukin-2 receptor beta chain, IL-2Rγ interleukin-2 receptor gamma chain, NK natural killer, PEG polyethylene glycol, PEG-IL-2 pegylated interleukin-2, PSTAT5 phosphorylated Signal Transducer and Activator of Transcription 5, Teff effector T cell, Treg regulatory T cell.

The structure of IL-2 bound to its heterotrimeric receptor complex^13,14^ has shown that the IL-2Rα chain (CD25) engages IL-2 distal to the IL-2Rβγ receptor subunits. Our previous studies employed the same semi-synthetic genetic code technology used in the current work to generate IL-2 variants intended for immuno-oncology indications with targeted PEG modifications on IL-2 at its interface with the IL-2Rα subunit of the receptor to block IL-2Rα engagement. This work led to the identification of SAR444245 (THOR-707) as a potent and specific activator of the IL-2Rβγ complex^39^. For AI diseases, we hypothesized that similar replacement of IL-2 residues in or near the IL-2Rβ or γ interfaces of IL-2 with nAAs followed by pegylation of these sites could produce a modified IL-2 with the reverse pharmacologic specificity: namely, a half-life extended pegylated IL-2 compound with reduced IL-2Rβγ interactions that requires IL-2Rα engagement for signaling (Fig. 1).

To identify residues that, when pegylated, specifically modulate the potency of IL-2 for the IL-2Rβγ complex, we selected 16 IL-2 residues in or near the IL-2Rβ and γ interfaces for production and functional analysis. Some of these amino acid sites, including V91, N88, and H16, were previously described^29,31,33^. IL-2 variants with the AzK substitutions in the designed residues were expressed as inclusion bodies in the *E coli-*based semi-synthetic organism strain, purified, and refolded (for full details, see ref. ^39^). The refolded AzK-modified IL-2 variants were then site specifically and covalently pegylated at the AzK residue using click chemistry via dibenzocyclooctyne (DBCO)-functionalized PEG molecules.

The resulting compounds were screened for differential receptor specificity and potency in primary immune cell subpopulations using multi-color flow cytometry and compared to previously described muteins and pegylated benchmarks (Supplementary Table 2). Potency was measured in fresh PBMC samples treated with either rhIL-2, mutein, or pegylated IL-2 variants. After incubation for 45 min, samples were stained with antibodies to detect the phosphorylated form of the transcription factor STAT5 (pSTAT5), a receptor-proximal marker of IL-2 receptor signaling, and a panel of surface markers to follow signaling and activation in specific lymphocyte subpopulations.

The results of the ex vivo profiling identified pegylated IL-2 compounds with a large range of potencies for Treg, CD8 + T, and NK cells (Supplementary Table 2). All pegylated variants showed measurable reduction in potency compared to the unmodified rhIL-2 compound, consistent with previous, attachment site independent and non-specific reduction in potency mediated by attached PEG^39^. On Tregs, the target cell population, pegylated variants showed a distribution of potencies that ranged from 2 to 15,000-fold reduction relative to native recombinant IL-2 protein (Supplementary Table 2). Compared to Treg potency, each variant showed similar or enhanced reductions in potency for the off-target CD8 + T and NK cell populations (Supplementary Table 2), which express little to no IL-2Rα. These results confirm that PEG modification of IL-2 near the IL-2Rβ and γ interfaces can modulate the potency of these compounds for stimulating different cell types, suggesting that the PEG modification may affect differences in receptor binding or assembly kinetics.

### In vivo screening of candidate compounds identified H16 as a pegylation site for selective stimulation of Tregs with minimal effects on CD8 + T and NK cells

**Fig. 2:**
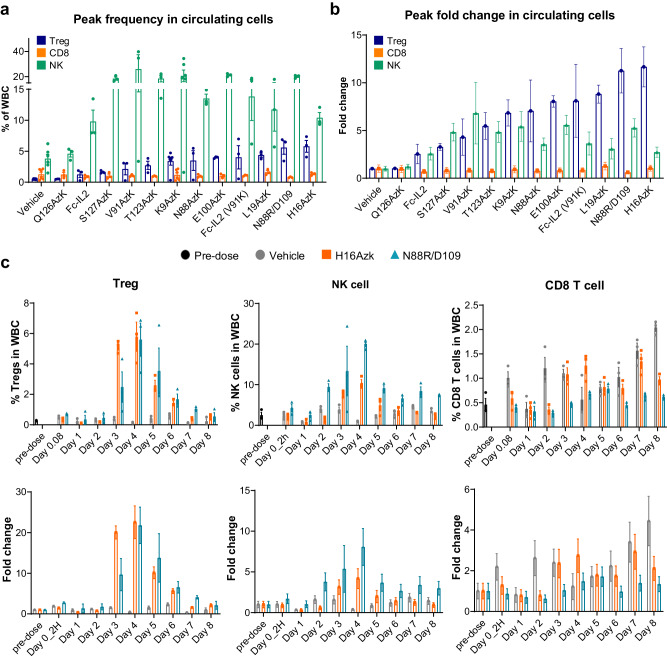
Compound screening in mice identified SAR’336, an IL-2 variant pegylated at residue H16, which significantly expanded Treg populations compared to other compounds. A single subcutaneous dose (~0.9 mg/kg) of the indicated variant was administered to C57BL/6 mice. Flow cytometry was used to quantitate Treg (CD4 + CD25+ FoxP3 +), CD8 T and NK cell expansion in peripheral blood. Shown in the plot is the (**a**) frequency and (**b**) maximum fold Treg expansion over vehicle for each compound (mean of three individual animals ± SEM); **(c)** Percentage and fold change of peripheral blood cells that were Treg, CD8 and NK cells is plotted as a function of time post dose (mean of three individual animals, ±SEM). Azk azide-containing non-canonical amino acid N6-(2-azidoethoxy)-carbonyl-L-lysine, CD4 cluster of differentiation 4, CD8 cluster of differentiation 8, CD25 cluster of differentiation 25, FoxP3 forkhead box protein 3, H16-Azk IL-2 variant with residue H16 replaced with the non-canonical amino acid Azk, IL-2 interleukin-2, NK natural killer cell, SEM standard error of mean, Treg regulatory T cell, WBC white blood cell.

While ex vivo profiling of pegylated IL-2 compounds identified molecules that exhibited a large range of potencies on target Treg cells, these data did not allow assessment of how potency differences translate to in vivo pharmacologic activity. To elucidate the potential interplay between ex vivo potency and in vivo PK and PD effects, we performed an in vivo screen comparing the capacity of each compound to specifically target Treg populations in mice. After a single intravenous dose (0.9 mg/kg) of each compound administered to C57BL/6 mice, peripheral blood samples were collected. Comparison of maximal Treg expansion revealed that IL-2 with residue H16 replaced with AzK and pegylated with a 30 kDa mPEG exhibited the highest change in peripheral Treg frequency (Fig. 2a, b). In contrast, Q126 did not alter the frequency in peripheral Tregs (Fig. 2a) despite having similar potency to H16 in vitro (Supplementary Table 2). These results indicate that in vitro potency is not a good indicator of Treg expansion, as measured by frequency of all cells. The percentage of Treg, CD8 T cells, and NK cells present in peripheral blood cells after treatment with H16 or N88R/D109 is plotted as a function of time post-dose in Fig. 2c. While H16 or N88R/D109 promoted strong expansion of Tregs in the periphery, minimal to low expansion of CD8 T cells or NK cells was observed with H16 treatment (Fig. 2c). In contrast, Fc-IL-2 and pegylated IL-2 molecules at S127, V91, T123, and K9 promoted similar fold-change expansion in frequency of peripheral Tregs and NK cells. Since this analysis focuses on cells within the periphery, it could exclude any potential differences that may exist in recruitment of immune cells into tissue by these variants.

Comparison of PK profiles of each candidate compound demonstrated that H16 exhibited an extended half-life with exposure among the longest in the set of compounds screened (Supplementary Fig. 2), followed by Q126. Interestingly, compounds of higher potencies for Tregs and other cell types, including the IL-2 molecule pegylated at residue L19, generally showed faster clearance rates and lower exposure compared to compounds of lower potency, including H16 (Supplementary Table 2).

### H16 induces expression of markers that correlate with Treg identity, activation, and suppressive function in mice

**Fig. 3:**
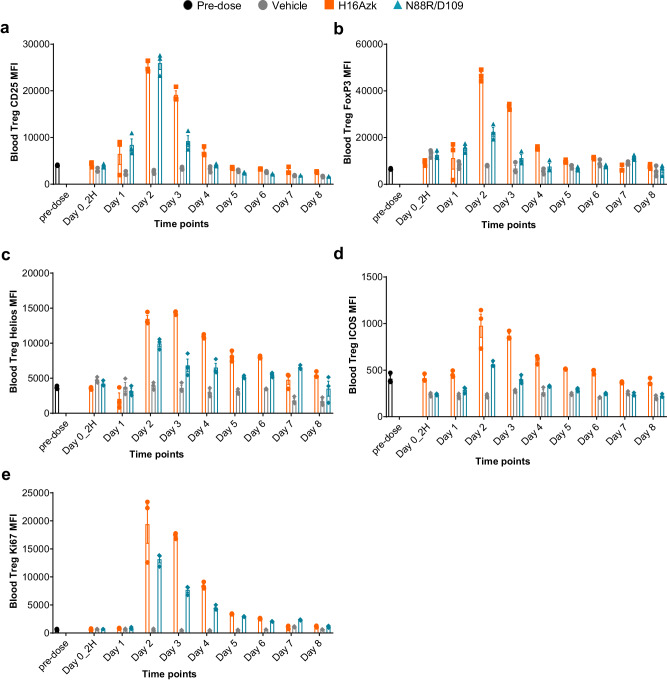
H16 administration stimulates increased Treg expression of markers that correlate with Treg identity, activation and suppressive function. H16 (0.9 mg/kg) was administered via tail vein injection to C57BL/6 mice, and peripheral blood was recovered at intervals post dose. Multi-color flow cytometry was used to profile individual cell populations. The expression of CD25 (**a**), FoxP3 (**b**), Helios (**c**), ICOS (**d**), and Ki67 (**e**) are shown (mean ± SEM for three individual animals at each point). CD25 cluster of differentiation 25, FoxP3 forkhead box protein 3, ICOS inducible T-cell co-stimulator, Ki67 Kiel 67, (proliferation marker), MFI median fluorescence intensity, SEM standard error of mean, Treg regulatory T cell.

As H16 demonstrated a superior Treg expansion and PK profile, we examined whether administration of this compound stimulated differential expression of markers that correlate with Treg function. Post-administration serial peripheral blood samples drawn from C57BL/6 mice described above were analyzed using flow cytometry for expression of markers that correlate with Treg activation and differentiation, including IL-2Rα (Fig. 3a), FoxP3 (Fig. 3b), Helios and ICOS, proteins known to be critical for Treg identity and suppressive function, as well as the proliferation marker Ki 67 (Fig. 3c–e). After H16 administration, the intensity of functional marker expression began increasing starting approximately one day post-dose, and expression levels peaked between day 2 and 3 (Fig. 3). CD25, FoxP3 and Ki67 signals were substantially increased over pre-dose levels, showing a 4- to 5-fold increased expression at their peak and decreased to near baseline levels by day 4 post-dose (Fig. 3a, b, and e). Signals for ICOS and Helios showed similar magnitudes of induction, with an approximately 3- to 4-fold increase over peak levels at day 2-3, albeit with a prolonged maintenance of expression levels and gradually decreasing levels to baseline after approximately 7 days (Fig. 3c, d). Together, these results show that H16 administration in mice drives Treg activation and upregulation of markers associated with proliferation and suppressive capacity.

### H16 drives dose-dependent Treg expansion and expression of markers of differentiation and suppressive function in non-human primate (NHP)

**Fig. 4:**
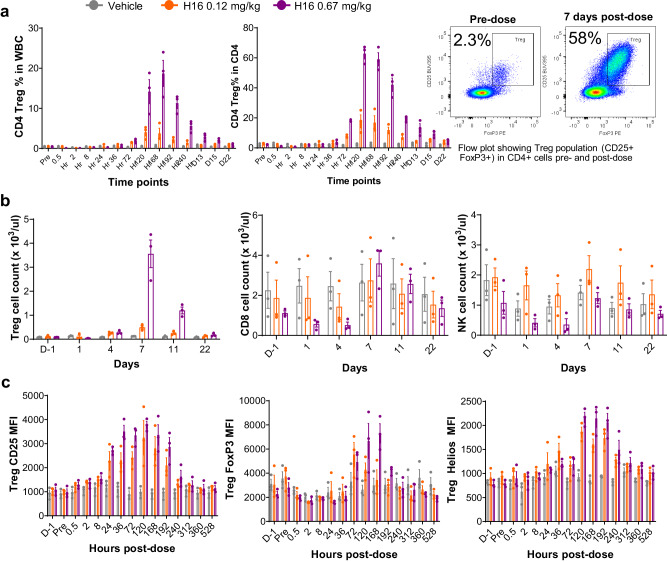
H16 induces dose-dependent, large-scale Treg expansion and expression of biomarkers of differentiation and suppressive function in NHP. **a**, **b** H16 administration to NHP drives Treg expansion in a dose-dependent manner. H16 was administered at 0 (vehicle only), 0.12 or 0.67 mg/kg to cynomolgus monkeys, and peripheral blood samples were collected at the indicated times. Multi-color flow cytometry was used to quantify (**a**) the percent of CD4+ Treg in WBC and CD4 T cells, and (**b**) cell counts for the CD4+ Treg, CD8 and NK cell populations by multiplying their frequency by the WBC counts. (**c**) H16 administration promotes the upregulation of biomarkers of CD4+ Treg differentiation and suppressive function. The median fluorescence intensity (MFI) as a function of time post-administration of H16 (0.12 mg/kg, orange; 0.67 mg/kg, purple) or vehicle (gray) is shown for CD25 (left panel), FoxP3 (middle panel), and Helios (right panel). Data are presented as mean ± SEM from three animals. CD4 cluster of differentiation 4, CD25 cluster of differentiation 25, FoxP3 forkhead box protein 3, MFI median fluorescence intensity, NHP non-human primate, NK natural killer, nonTreg non regulatory T cell, rhIL2 recombinant human interleukin-2, SEM standard error of mean, Treg regulatory T cell, WBC white blood cell.

To study the effect of dose level on peripheral exposure and PD, increasing doses of H16 were administered to cynomolgus monkeys and peripheral blood samples were collected and analyzed by flow cytometry. The results demonstrated that, at increasing dose, the exposure increased proportionally (Supplementary Fig. 3a) and CD4+ Tregs as a percentage of total peripheral blood cells (Fig. 4a, Supplementary Fig. 3b) or as a percentage of CD4 T cells (Fig. 4a, Supplementary Fig. 3c) showed large increases the populations. At the highest dose evaluated (0.67 mg/kg), Treg frequency peaked at approximately 20% of whole blood and 60% of CD4 T cells (Fig. 4a). Increased frequency of CD4 Tregs (Fig. 4a) correlated with an increase in total cells in the circulation (Fig. 4b). Minimal expansion of CD8 + T and NK (Fig. 4b) populations was observed at doses of H16 that drove substantial expansion of Treg populations. Similarly to mouse, administration of H16 promoted Treg upregulation in the intensity of CD25, FoxP3, Helios, and pSTAT5 (Fig. 4c, Supplementary Fig. 3d), which correlate with differentiation and Treg suppressive function, and increased levels of circulating soluble CD25 were observed (Supplementary Fig. 3e), which peaked 96–120 h post-dosing.

### H16 extension of peripheral half-life and activation of Tregs in NHP is modulated by PEG length

**Fig. 5:**
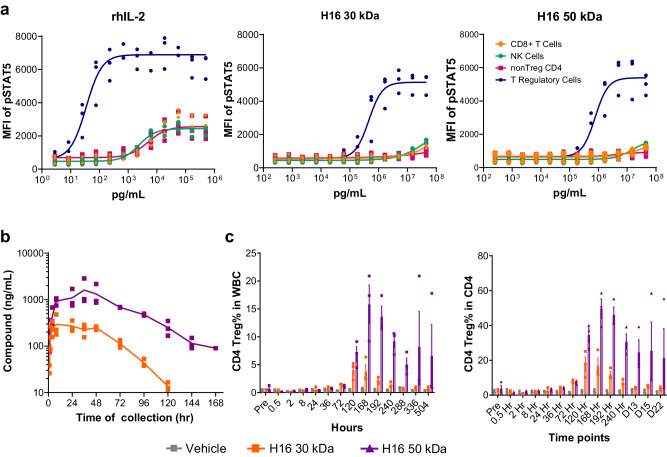
PEG length modulates H16 exposure and pharmacodynamics in NHP. **a** Pegylation of H16 with 30 kDa or 50 kDa linear mPEG retains bias towards signaling in Treg ex vivo. NHP PBMC were stimulated in triplicate with concentration series of rhIL-2, H16-30 kDa or H16-50 kDa. Treated cell populations were analyzed using multi-color flow cytometry to detect and quantify pSTAT5 in different cell subsets. The plots shown represent the average pSTAT5 fluorescence intensity, fit to a baseline restrained 4 parameter logistic regression and normalized to maximum signal to facilitate comparison of potency, with error bars representing SEM. **b**, **c** A single subcutaneous dose, estimated to reach EC50 at the target Treg cell, of H16-30 kDa (orange) or H16-50 kDa (purple) was administered to cynomolgus monkeys. **b** Peripheral blood samples were collected at the indicated times post-dose and analyzed for H16 level using ELISA (see *Methods*). **c** Peripheral blood samples were collected at the indicated times, and multi-color flow cytometry was used to quantify Treg (CD4 + CD25+ FoxP3 +) expansion; data shown as Treg percent in WBC and CD4 + T cells (total peripheral blood cells at each time point; data points represent the mean from three individual animals with error bars corresponding to SEM. CD4 cluster of differentiation 4, CD8 cluster of differentiation 8, CD25 cluster of differentiation 25, EC50 half-maximal effective concentration, ELISA enzyme-linked immunosorbent assay, FoxP3 forkhead box protein 3, kDa kilo Dalton, MFI median fluorescence intensity, NHP non-human primate, NK natural killer, PBMC peripheral blood mononuclear cells, PEG polyethylene glycol, pSTAT5 phosphorylated Signal Transducer and Activator of Transcription 5, SEM standard error of mean, Treg regulatory T cell, WBC white blood cell.

To test the effect of PEG length on H16 pharmacology, H16 was conjugated to either a 30 kDa or 50 kDa linear mPEG. In vitro, H16 conjugation to a larger size PEG polymer (50 kD versus 30 kD) resulted in a modest reduction in potency ex vivo (Fig. 5a). When administered to NHPs, PEG50 conjugates of H16 showed significantly extended half-life (Fig. 5b) and exposure measured as area under the curve (Table 1), promoting a surprisingly greater peripheral Treg expansion compared to 30 kD pegylated H16 (Fig. 5c). Thus, all further studies in the NHP model were conducted using the H16 variant bioconjugated with PEG 50 kD, hereafter referred to as the clinical candidate SAR’336.

**Table 1:** PK profile of H16 conjugated to 30 and 50 kDa PEG in NHP following SC administration

Variant	NHP #	AUC_last_/dose (h*kg*ng/mL/mg)	C_max_/dose (kg*ng/mL/mg)	t_1/2_ (h)
H16 30 kDa	1	183,000	3,990	17.6
	2	133,000	2,200	16.8
	3	142,000	2,840	17.0
H16 50 kDa	1	410,000	4,710	28.8
	2	429,000	5,300	27.0
	3	728,000	14,300	28.1

Increasing the dose of SAR’336 from 0.0025 to 0.2 mg/kg in NHP induced large-scale increases in the frequency of peripheral Tregs in blood in a dose-dependent fashion, in all instances, peaking at approximately 1-week post-dose (Supplementary Fig. 4). Similarly to H16 bioconjugated to a 30 kD PEG (Fig. 4b), SAR’336 promoted Treg expansion with minimal changes in CD8+ or NK cell frequencies. Quantification of the induction of biomarkers for activation and proliferation in peripheral blood revealed robust induction of pSTAT5, Ki-67, and CD25. Similarly, levels of FoxP3 and Helios, markers that correlate with differentiation and suppressive function of Tregs in peripheral blood, were also increased (Supplementary Fig. 4).

### SAR’336 engages IL-2Rα with high affinity, but does not measurably interact with IL-2Rβ

**Fig. 6:**
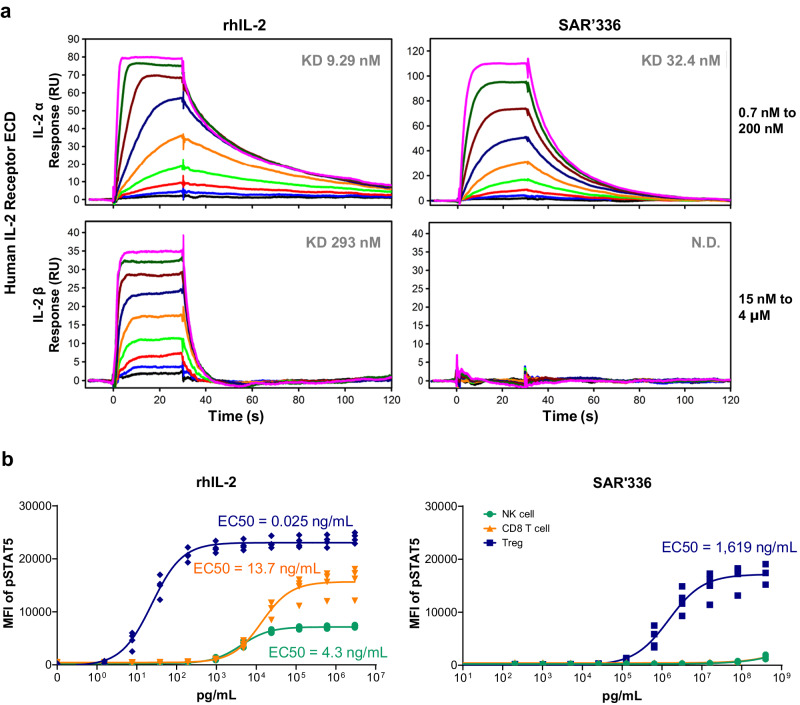
SAR’336 engages IL-2Rα with high affinity but does not measurably interact with the IL-2Rβ subunit. **a** Biochemical characterization of SAR’336 interactions with IL-2 Rα and β receptor subunits using SPR demonstrates that SAR’336 engages the IL-2Rα with high affinity but does not measurably engage the IL-2Rβ subunit. Extracellular domains of the human IL-2Rα (top row) and β (bottom row) subunits were immobilized on the surface of a SPR sensor and probed with a dilution series of either rhIL-2 (left column) or SAR’336 (right column). Test samples were injected for 60 s to allow association, followed by buffer only to measure dissociation kinetics. Response units (RU, Y-axis) are plotted versus time (s, X-axis). The colors correspond to each test concentration as shown (inset). **b** Assessing pSTAT5 levels SAR’336 in human primary lymphocytes shows that SAR’336 potency on Tregs is reduced compared to IL-2, but highly specific for Treg signaling versus CD8 + T cells and NK cells. Human PBMC samples from 4 healthy donors were stimulated with concentration series of rhIL-2 or SAR’336. Treated cell populations were analyzed using multi-color flow cytometry to detect and quantify pSTAT5 levels in different cell subsets. The plots shown represent the average (+ SEM) pSTAT5 fluorescence intensity, fit to a baseline restrained 4 parameter logistic regression and normalized to maximum signal to facilitate comparison of potency on Treg, NK and CD8 + T cell subsets. Average EC50 values are shown. Individual EC50 values are reported in Supplementary Table 4. CD8 cluster of differentiation 8, EC50 half-maximal effective concentration, ECD extracellular domain, IL-2 interleukin-2, IL-2 Rα interleukin-2 receptor alpha, IL-2 Rβ interleukin-2 receptor beta, KD dissociation constant, MFI median fluorescence intensity, ND not determined, NK natural killer, PBMC peripheral blood mononuclear cells, rhIL-2 recombinant human interleukin 2, RU response units, pSTAT5 phosphorylated Signal Transducer and Activator of Transcription 5, S seconds, SEM standard error of mean, SPR surface plasmon resonance, Treg regulatory T cell.

As the pegylation site in SAR’336 replaces a residue that makes direct contact with the IL-2Rβ subunit at the IL-2/IL-2Rβ interface, we hypothesized that this modification may cause differential binding kinetics to the IL-2Rβ subunit. To understand the mechanism by which SAR’336 demonstrates a bias for stimulation of Treg proliferation and activation over CD8 + T and NK cells in NHP, we used SPR to directly measure binding. The extracellular portions of the human IL-2Rα and β were immobilized on the surface of SPR sensor chips, and concentration series of rhIL-2 or SAR’336 were applied to these surfaces. Binding to the receptors was monitored as the change in response units as a function of time during association and dissociation (wash) phases. On IL-2Rα surfaces, rhIL-2 showed high affinity binding characterized by rapid association and slow dissociation kinetics (Fig. 6a, **upper left panel**, Supplementary Table 3). Similarly, SAR’336 interacted with high affinity with the IL-2Rα surface, with an approximately 3-fold reduction in affinity as expected for non-specific PEG effects on binding (Fig. 6a, **upper right panel**, Supplementary Table 3). In contrast, surfaces containing immobilized IL-2 Rβ showed robust association and dissociation responses with rhIL-2 (Fig. 6a, **lower left panel)**, while SAR’336 did not measurably engage the IL-2Rβ subunit surface, even at high concentrations (Fig. 6a, **lower right panel**). These results suggest that SAR’336 retains interaction with the IL-2Rα subunit but lacks any measurable affinity to the IL-2Rβ subunit.

### SAR’336 specifically activates pSTAT5 expression in Tregs but not CD8 + T cells ex vivo

To determine how the reduced IL-2Rβ affinity of SAR’336 affects activation of primary human immune cell subpopulations, we profiled the effect of this engineered cytokine on lymphocyte activation in human PBMC samples. In Treg populations, rhIL-2 showed potent stimulation of pSTAT5 formation compared to SAR’336 (Fig. 6b, Supplementary Table 4), suggesting that a slight reduction in IL-2Rα affinity and absence of IL-2Rβ engagement may significantly affect SAR’336 signaling compared to rhIL-2. In CD8 + T and NK cell populations, SAR’336 was not observed to stimulate pSTAT5 phosphorylation (Fig. 6b), unlike rhIL-2. With rhIL-2, potency in NK cells is 120 - 250-fold less than that observed in Tregs. However, a similar ratio of pSTAT5 induction in NK cells and CD8 T cells was not observed with SAR’336, suggesting that SAR’336 may be both reduced in overall potency and more specifically deficient in stimulation of the IL-2 βγ receptor complex in CD8 and NK cells that lack IL-2 Rα. Taken together, these results suggest that SAR’336 is strongly reduced for signaling potency at the IL-2 Rβγ complex, but that this reduction in potency seems to be overcome by binding to the IL-2Rα subunit that is highly expressed on Tregs.

### SAR’336 treatment reduces inflammation in mouse DTH model

**Fig. 7:**
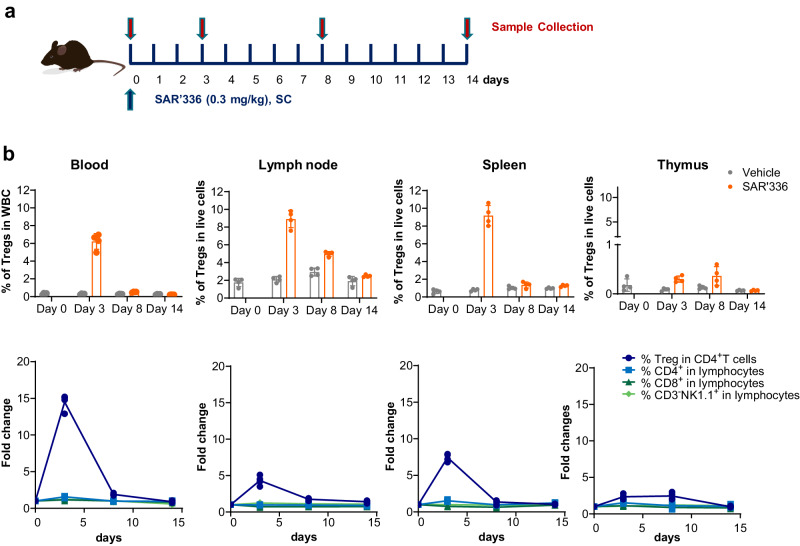
SAR’336 induces Treg expansion in the blood and lymphoid organs in mice. **a** C57BL6 mice were treated with 0.3 mg/kg SAR’336 subcutaneously. **b** Frequency of CD4+ Treg cells in blood, lymph nodes, spleen, and thymus was measured. Groups of 10-week-old C57BL6 mice (n = 4) were given a single dose of SAR’336 subcutaneously at 0.3 mg/kg or vehicle. Blood, spleen, and lymph nodes were collected at 3-, 8- or 14-days post dose. Flow cytometry was performed on whole blood and disaggregated cells from tissues to assess relative levels of Treg (CD4 + , CD25 + , FoxP3 +), CD4, CD8 and NK populations. Fold change was compared to an untreated baseline group (n = 4). CD3 cluster of differentiation 3, CD4 cluster of differentiation 4, CD25 cluster of differentiation 25, FoxP3 forkhead box protein 3, NK natural killer, SC subcutaneous, Treg regulatory T cell, WBC white blood cell. Mouse vector illustration credit: [baluchis]/stock.adobe.com.

**Fig. 8:**
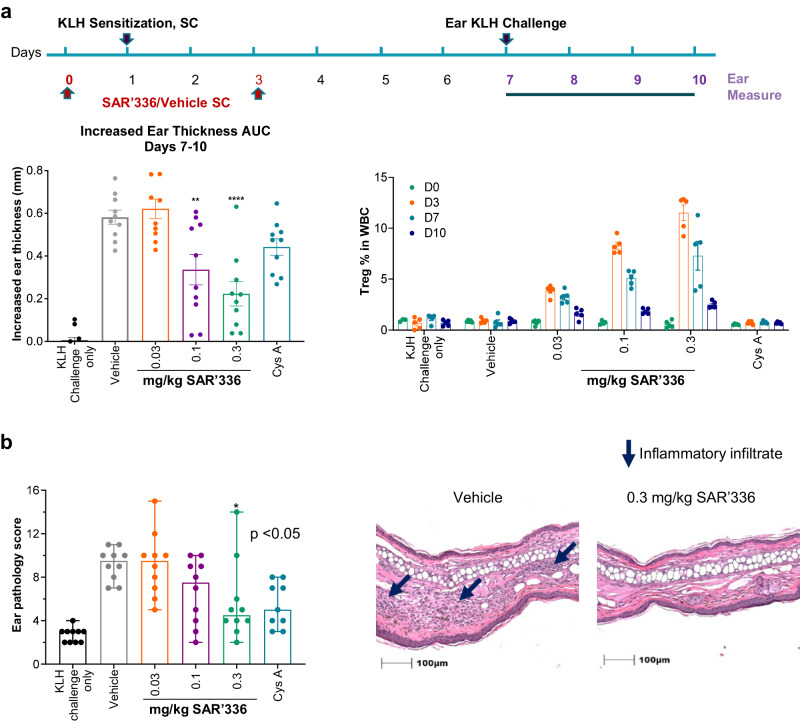
SAR’336 reduces inflammation in a mouse DTH model. Groups of C57BL6 mice (n = 10) were sensitized with 250 µg KLH with CFA/IFA on Day 1 and dosed on day 0 & 3 with SAR’336 at 0.03, 0.1 & 0.3 mg/kg. Ear pinnae were challenged with 10 µg KLH on day 7 and ear thickness readings were measured on days 7-10. **a** Treg cells were measured in blood on days 0, 3, 7 & 10, showing dose-dependent induction. Overall ear thickness from days 7-10 was significantly lower in 0.1 & 0.3 mg/kg dose groups (t test, p < 0.002, 0.0001) compared to vehicle. **b** Ear pathology was assessed by scoring inflammatory infiltrates, hyperkeratosis, epidermal hyperplasia, and corneal pustules, which were compiled into an overall score, showing that the 0.3 mg/kg group improved vs vehicle (1-way ANOVA p < 0.05). Representative images show reduced inflammatory infiltrates with SAR’336 treatment. ANOVA analysis of variance, AUC area under the curve, CFA complete Freund’s adjuvant, D day, IFA incomplete Freund’s adjuvant, KLH keyhole limpet hemocyanin, SC subcutaneous.

To confirm that the PD and pharmacological effects of SAR’336 administration were not limited to the periphery, C57BL/6 mice were administered either the vehicle or SAR’336 (0.3 mg/kg, SC administration) and peripheral blood samples and lymphoid organs were collected on days 0 (baseline) and 3-, 8-, and 14-days post-treatment (Fig. 7a). SAR’336 induced higher frequencies of Treg cells in blood as well as secondary lymphoid organs compared to vehicle (Fig. 7b). After a single dose, 5-11% of cells in the circulation, lymph node, and spleen were CD25+FoxP3+ Tregs, which translates to a synchronized 5- to 15-fold increase in the percentage of Tregs after 3 days, and a lower increase in Treg frequency in the thymus peaking on day 8 (Fig. 7b). In a mouse ear model of KLH-induced DTH, repeat dosing of SAR’336 induced dose-dependent increase in Treg frequency that peaked on day 3, the day of the second repeat dose, and remained elevated 10 days after the first dose (Fig. 8a). At 0.1 and 0.3 mg/kg dose, SAR’336 significantly suppressed the amount of ear swelling (Fig. 8a) as well as decreased inflammatory cell infiltration into ear tissue (Fig. 8b) at 0.3 mg/kg dose, following re-challenge with KLH.

### Tregs treated with SAR’336 are potent suppressors of CD4+ and CD8 + T cell proliferation ex vivo

**Fig. 9:**
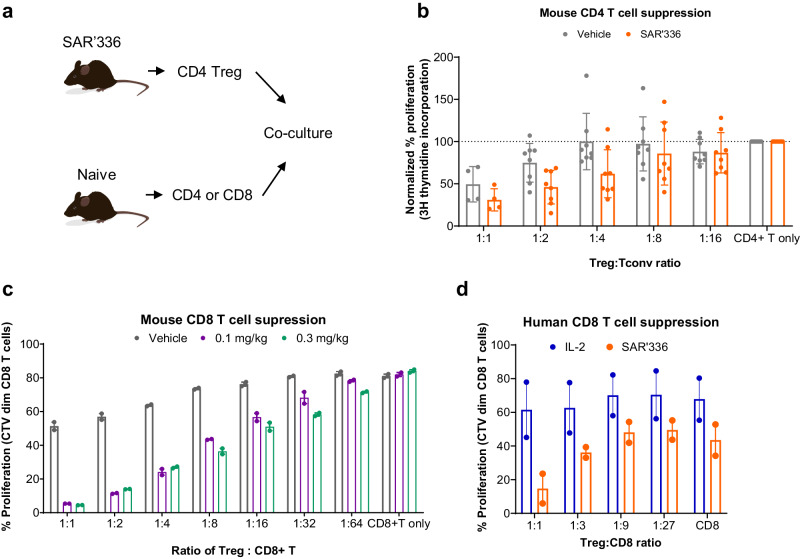
Tregs treated with SAR’336 are potent suppressors of CD8 + T cell proliferation ex vivo. **a** Schematic of the experiment to measure ex vivo suppressive capacity of Tregs: mice were administered either vehicle or SAR’336 (0.3 mg/kg), and splenocyte populations were collected at 2 days post-dose. The CD4+ Treg cells were isolated from the splenocytes derived from dosed and naïve animals. Next, CD4 or CD8 + T cells were harvested from splenocytes from naïve or vehicle-treated animals. To measure ex vivo suppressive capacity, Tregs were co-cultured at different ratios with CD4 or CD8 + T cells from naïve or vehicle-treated animals. After 3 days of co-culture, the (**b**) CD4 + T cells and (**c**) CD8 + T cell populations from each condition were analyzed for proliferation using thymidine incorporation (**b**) or flow cytometry (**c**); [mean ± SD]. **d** CD8 + T cells and Tregs were sorted from freshly isolated PBMC and cultured 4 days with recombinant IL-2 or SAR’336. Proliferation of CD8 T cells is reported. N = 2 donors pooled from independent experiments. Symbols represent individual donors. Lines represent mean response. CD4 cluster of differentiation 4, CD8 cluster of differentiation 8, CTV CellTrace Violet™ (dye), conv conventional T cell, Treg regulatory T cell. Mouse vector illustration credit: [baluchis]/stock.adobe.com.

As SAR’336 administration in mice stimulated the large-scale expansion of Treg populations, upregulation of markers that correlate with activity, and in vivo suppression of the DTH response, we hypothesized that the SAR’336-treated Treg populations have enhanced suppressive capacity compared to Tregs from naïve animals. To test this hypothesis, mice were treated with either vehicle or SAR’336, then splenocyte populations were collected at 2 days post-dose, harvesting CD4+ Treg cells from SAR’336-treated and vehicle-treated animals. Next, CD4+ or CD8+ conventional T (Tconv) cells were harvested from splenocytes of naïve animals and labeled with CTV dye or 3H-thymidine, and co-cultured at different ratios with Tregs derived from naïve or SAR’336-treated animals (Fig. 9a). After co-culture, the CTV-labeled effector T cell population from each condition was analyzed for proliferation using flow cytometry. Tregs from naïve animals showed the capacity to suppress the proliferation of CD4+ and CD8+ cells, but Tregs from SAR’336-administered mice demonstrated higher suppressive capacity at similar Treg:Tconv ratios, indicating that SAR’336-treated Tregs are superior suppressors on a per-cell basis (Fig. 9b, c). Similar co-culture of human CD8 T cells with Tregs in the presence or absence of SAR’336 confirmed these findings (Fig. 9d). These results suggest that SAR’336 effectively induces Tregs that have a significantly higher functional suppressive capacity.

## Discussion

While study of the human cytokine IL-2 has been an area of intense focus in therapeutic development, efforts to utilize IL-2 and other cytokines for the treatment of immunologic disease has been challenging due to complex and pleiotropic signaling effects. In the case of IL-2, this cytokine displays dual functionality via both induction of immune responses through stimulation of proliferation of effector T (Teff) and NK cells, and control of immune responses through maintenance of Treg cells^3–5,11–14^. These dual activities are governed by, and inherently differ from, each other on the basis of the concentrations of IL-2 available, its unique binding affinities for the individual components of the receptor complex, and their subsequent cell-specific expression^3–5,11–14,16^. These challenges have presented opportunities for new technologies to engineer pharmacologically fine-tuned IL-2 variants with enhanced therapeutic properties^2,5,8–10^.

Seminal studies employed non-specific or semi-specific methods to pegylate cytokines in an effort to bias receptor-specific potency and identified multiply-pegylated protein variants with improved specificity for a receptor complex of interest^40–43^. Later studies applied similar methodologies to IL-2 in an effort to bias IL-2 stimulation of Tregs^35^. These efforts lacked sufficient precision or functional generality to identify single pharmacologically improved species. Our previous work employed the semi-synthetic expanded genetic code technology platform from Synthorx to engineer pegylated IL-2 SYNTHORIN™ compounds with superior effector-stimulatory specificity via ablation of interaction with the IL-2Rα^39^. In this manuscript, we demonstrate that this technology can enable a more sophisticated modulation of receptor binding, using PEG positioning-specific effects to identify variants with reduced engagement of a receptor subunit, yet allowing receptor complex formation and signaling. Our approach employed the semi-synthetic organism platform using a novel, synthetic DNA base pair (X-Y) to generate a set of candidate compounds in which stable PEG molecules were covalently attached to novel nAAs installed at distinct sites within the cytokine with the aim of modulating affinity at the IL-2Rβγ chain of the IL-2 receptor without major effects on affinity for the IL-2Rα chain. Functional screening of a library of such compounds via Treg expansion in mice identified H16, a rhIL-2 variant with the natural residue replaced with the nAA, AzK and pegylated, as a candidate with enhanced capacity to expand and activate peripheral Tregs without activation of CD8 + T cells and NK cells. Further optimization of the molecule with conjugation of a larger 50kDa PEG at H16, lead to the identification of SAR’336. Administration of SAR’336 induced biomarkers of Treg differentiation and suppressive function, including FoxP3, in mouse and NHP models. Dose-dependent increases were also seen in the T cell activation marker ICOS and the transcription factor Helios, which are expressed at high levels in Treg cells and correlate with stability of the Treg cell phenotype and suppressive function.

Our data demonstrate a medicinal chemistry-like methodology for discovery of biologic drugs with novel properties. While previous studies have identified natural residue substitutions, including residues at H16 and N88 that modulate IL-2 potency to the IL-2 receptor beta subunit^29,31–33^, our pegylation strategy enabled significantly differentiated properties to be identified in vitro and in vivo. The first step in this process was identification of residues that, when pegylated with a 30 kDa PEG modification, adjusted the cell-specific activity and potency of the resulting molecule. When the H16 variant of IL-2 was conjugated with a 50 kDa linear PEG, the larger conjugate was modestly reduced in potency (approx. twofold reduction in EC_50_, the half-maximal effective concentration) relative to a 30 kDa PEG, as determined by pSTAT5 activation in Treg cells. However, a single SC dose of H16 with a 50 kDa PEG administered to cynomolgus monkeys unexpectedly produced a much larger exposure relative to the 30 kDa PEG bioconjugate (3.4X). SAR’336 with a 50 kDa PEG also induced a larger and more persistent expansion of peripheral Treg cells in NHP relative to the 30 kDa PEG bioconjugate. We conclude that peripheral Treg expansion in response to SAR’336 is exposure-driven, with the size the of PEG bioconjugate likely limiting renal filtration and improving both compound exposure and levels of Treg expansion.

Our data show that SAR’336 interactions with the IL-2Rβ subunit are too weak to be measured using SPR. However, the current models for IL-2 receptor signaling suggest that recruitment of the IL-2Rβ subunit is required for signaling^3–5,11–14^. We therefore hypothesize that high-affinity engagement of the IL-2Rα subunit by SAR’336 may allow presentation to the IL-2Rβ subunit to overcome this strong potency reduction and allow productive signaling complex assembly. However, it is also possible that SAR’336 engagement of the IL-2Rα chain may recruit IL-2Rβ and γ subunits via an atypical assembly pathway, potentially utilizing the modest affinity afforded by the IL-2Rβ and γ subunits^11^ to assemble a functional, albeit potency-reduced signaling complex.

Muteins of IL-2 with a decreased affinity for the IL-2Rβ chain have been developed to improve Treg specificity and disfavor the proliferation of Teff and NK cells and these have shown remarkable Treg selectivity^29,31–33^. However, typical residues targeted by such muteins occupy regions of the IL-2 protein that are enriched for P1 anchor sequences that allow peptide presentation on class II major histocompatibility complexes (MHCs). Indeed, initial variants that utilize these sites have generated anti-drug antibody (ADA) formation in human clinical trials, and we speculate that this may be a limitation of both the method and of the IL-2 variants that rely on this regional modification. While ADAs may limit the efficacy of a drug over time, muteins that drive potential anti-drug immune responses may further lead to epitope spreading and neutralization of the patient’s native protein^31,44,45^, further limiting the use of these methods. In contrast, the mutein-generating approach presented here utilizes chemically stable covalent PEG modification, which may avoid involvement of steps in the class II MHC presentation pathway and limit subsequent immunogenicity and ADA formation, resulting in an overall safer therapeutic agent. The lack of ADA formation in clinical studies of the SAR444245 molecule further supports this contention^46^.

Recently, Zhang et al.^36^ presented evidence of improved PK and immunosuppression by an engineered IL-2 molecule in mouse models of AI diseases by orthogonally conjugating with PEG via copper-free click reaction through incorporation of azide-containing amino acids using a canonical amber suppression method to incorporate AzK^47^. Unlike SAR’336, which contains a 50 kDa PEG at site H16, these authors utilized 20 kDa PEG conjugations at Y31 and T51 to achieve their desired phenotype. Using a different approach, NKTR-358 conjugated native rhIL-2 protein with PEG moieties using standard chemical coupling methodologies^35^. In a single-ascending dose study in healthy volunteers, NKTR-358 demonstrated a dose-dependent expansion of proliferating CD25+ Treg cells. No measurable changes in the numbers and percentages of CD4+ and CD8 + T cells were seen, but low-level increases in NK cell numbers were observed at the highest doses tested^48^. Interestingly, the NKTR-358 compound has been reported to feature significant reductions in IL-2Rα, as well as IL-2Rβ engagement, a substantial functional difference from SAR’336. We speculate that these differences may arise due to the non-specific pegylation chemistry used to generate the NKTR-358 molecule, which may target residues with undesired pharmacological consequences. Additional studies will be required to determine the differential and comparative pharmacology observed for these compounds compared to SAR’336.

In the widening field of cytokine therapies for AI disorders, precision engineering of biologics will enable increased specificity and reduced off-target pleiotropy to be designed into natural immune regulatory cytokines. The application of the semi-synthetic genetic code technology to introduce site-specific and orthogonal chemistry via novel amino acids to achieve targeted bioconjugation of moieties led to the identification and design of SAR’336, an improved, pegylated IL-2 with enhanced PK and a more specific target binding profile. The concepts and strategies utilized here to improve IL-2 have potential for future development of other biologic scaffolds that would benefit from the site-specific modulation of potency, MHC-II epitope protection, and receptor specificity afforded by our technology. Together, the findings presented here demonstrate that SAR’336 is a specific activator of Treg proliferation and suppressive function, with minimal expansion of Teffs and NK cells populations. These results from mouse and NHP models support the continued development of SAR’336 as a clinical development candidate for treatment of AI-related disorders.

### Supplementary information

Supplementary Information

Description of Additional Supplementary Files

Supplementary Data 1

Reporting Summary

## Data Availability

The datasets generated and/or analyzed during the studies reported herein are available from the corresponding author upon reasonable request. Source data for the figures are available as Supplementary Data 1.
