# Supplementary material for: A CD25-biased interleukin-2 for autoimmune therapy engineered via a semi-synthetic organism
Source: Commun Med (Lond). 2024 Mar 26;4:58. doi: 10.1038/s43856-024-00485-z (PMC10966033; doi:10.1038/s43856-024-00485-z)
Supplement: Supplementary file 1 — Supplementary Information [file 43856_2024_485_MOESM1_ESM.pdf]

SUPPLEMENTARY INFORMATION

A CD25-biased interleukin-2 for autoimmune therapy engineered via a semi-synthetic organism

Jerod L. Ptacin[1], Lina Ma[1], Carolina E. Caffaro[1], Nicole V. Acuff[1], Kristine Germar[2], Peter Severy[2], Yanyan Qu[2], Jose-Luis Vela[2], Xinming Cai[2], Kristine M. San Jose[1], Hans R. Aerni[1], David B. Chen[1], Ean Esche[1], Taylor K. Ismaili[1], Rob Herman[1], Yelena Pavlova[1], Michael J. Pena[1], Jasmine Nguyen[1], Lilia K. Koriazova[1], Laura K. Shawver[1], Ingrid B. Joseph[1], Jill Mooney[1], Mark Peakman[2], and Marcos E. Milla[1*]

[1]Synthorx, a Sanofi Company. 11099 N. Torrey Pines Rd. Suite 190, La Jolla, CA 92037

[2]Sanofi. 350 Water St, Cambridge, MA 02141, USA

[*]To whom correspondence should be addressed: marcos@samsaracap.com

Supplementary Figure 1. Representative gating strategy in (a) NHP and (b) mouse whole blood is shown.

(**a**) For NHP, NK cells are defined as CD14-CD20-CD3-CD7+, CD8 T cells as CD14-CD20-CD3+CD8+, non-Treg CD4 as CD14-CD20-CD3+CD4+CD25-FoxP3-, and Tregs as CD14-CD20-CD3+CD4+CD25+FoxP3+. (**b**) For mouse, NK cells are defined as CD19-GR1-NK1.1+, CD8 T cells as CD19-GR1-NK1.1-TCRb+CD8+, non-Treg CD4 as CD19-GR1-NK1.1-TCRb +CD4+CD25-FoxP3-, and Tregs as CD19-GR1-NK1.1-TCRb+CD4+CD25+FoxP3+

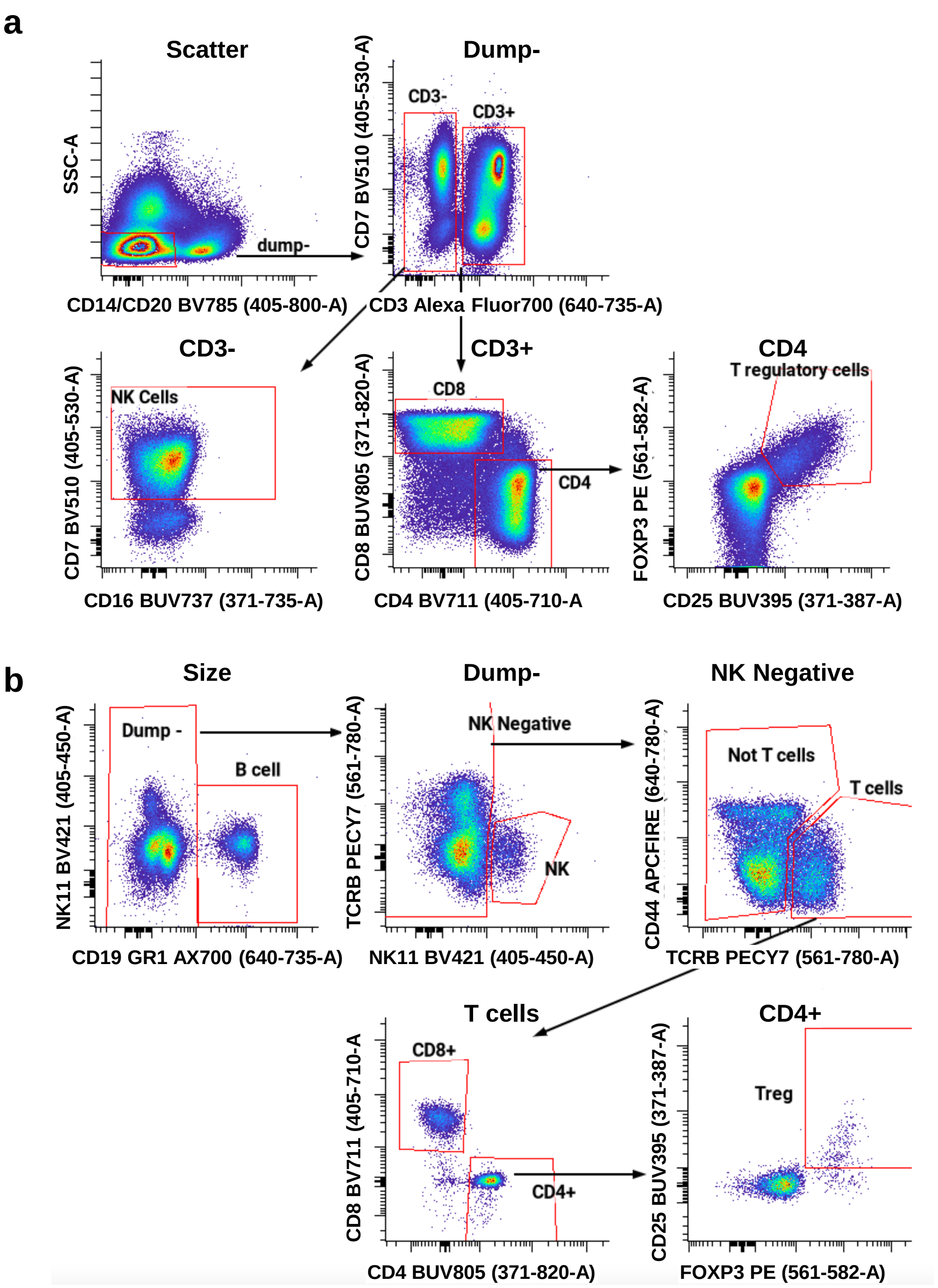

Supplementary Figure 2. PK profile of IL-2 variants in C57BL/6 mice.

Compound screening in mice identified H16 as a pegylated IL-2 variant which significantly expanded Treg populations compared to other compounds. A single subcutaneous dose of the indicated variant was administered to C57BL/6 mice. (**a**) Concentration is plotted as a function of time post dose. (N=3 ± SEM)

a

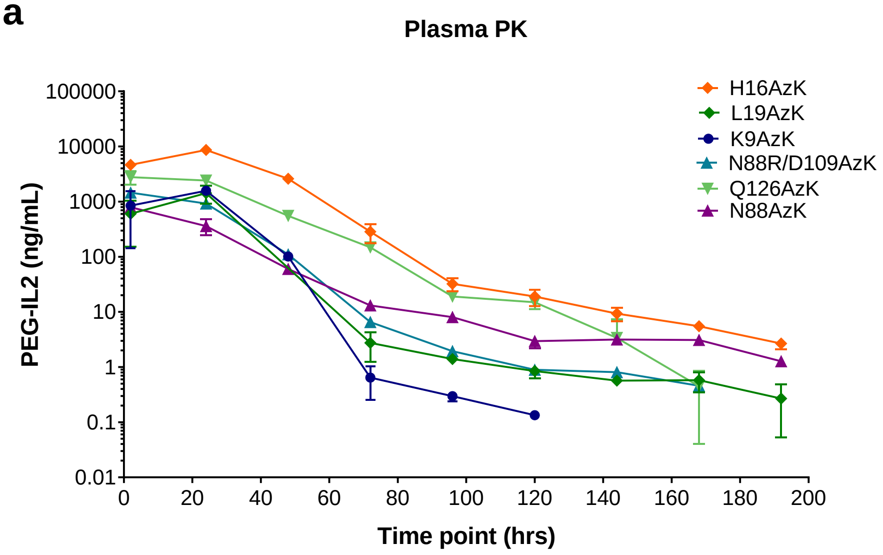

Supplementary Figure 3. H16 is a half-life extended IL-2 that promotes activation of Tregs in cynomolgus monkey.

H16 induces dose-dependent and large-scale Treg expansion and expression of biomarkers of differentiation and suppressive function in NHPs. (**a**) H16 was administered at 0.02, 0.07, or 0.2 mg/kg to cynomolgus monkeys. Pharmacokinetics of variants up to 240 hours post treatment. (**b-e**) H16 was administered at 0 (vehicle only), 0.07, or 0.2, mg/kg to cynomolgus monkeys, and peripheral blood samples were collected at the indicated times. Multi-color flow cytometry was used to quantitate the CD4+Treg as a **(b)** percentage of WBC **(c)** percentage of CD4 T cells, and **(d)** expression of pSTAT5. (At 0.2 mg/kg dose, day 3 time-point is excluded for poor sample collection). (**e**) Soluble CD25 (sCD25) was quantified from serum. Data represent mean ± SEM from three independent animals.

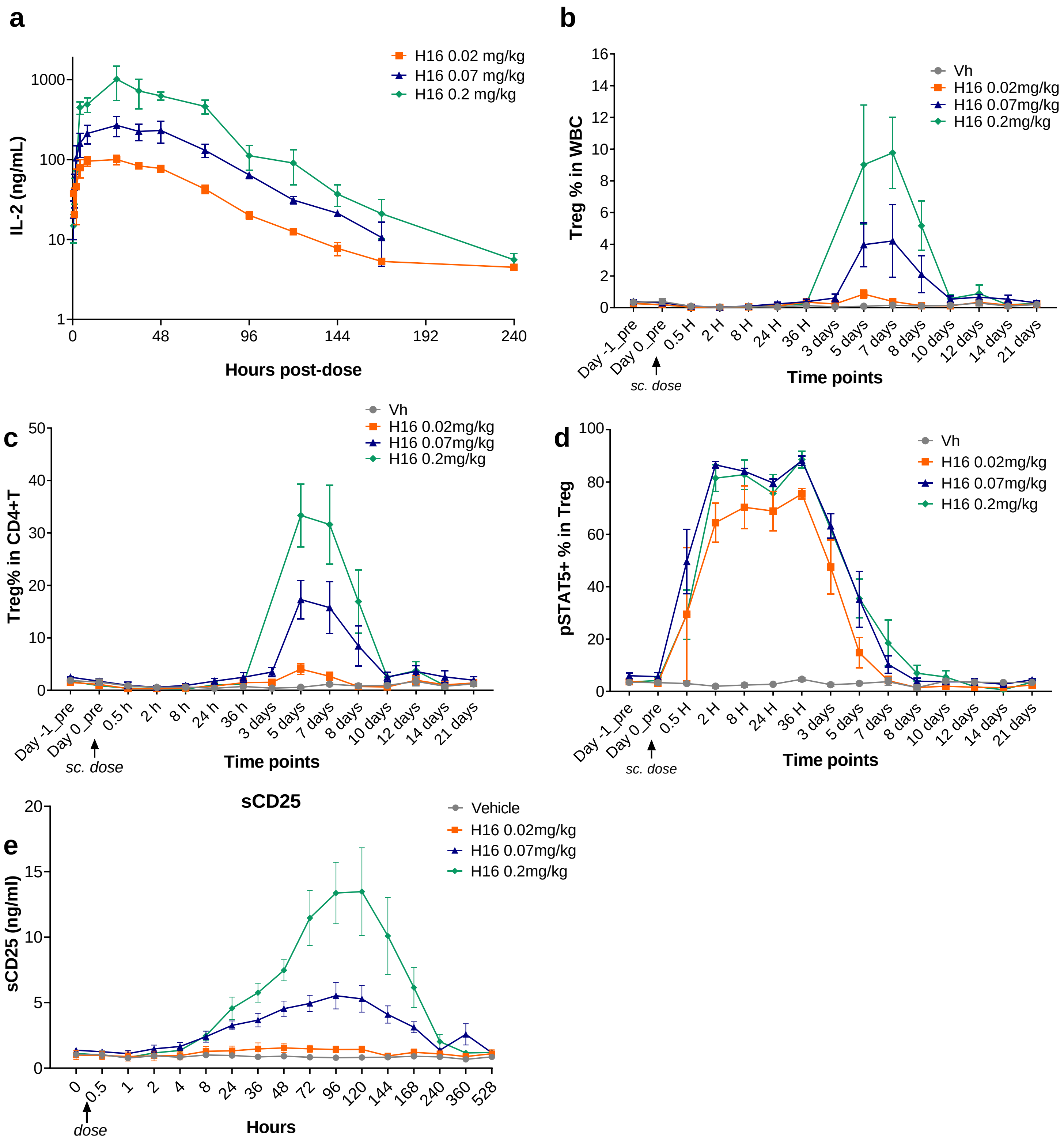

Supplementary Figure 4. SAR'336 promotes dose dependent Treg expansion and activation in NHP.

SAR'336 induces dose-dependent and large-scale Treg expansion and expression of biomarkers of differentiation and suppressive function in NHPs. THOR-809 was administered at 0.0025 – 0.2 mg/kg to cynomolgus monkeys and N88R/D107 at 0.3 mg/kg, and peripheral blood samples were collected at the indicated times. Multi-color flow cytometry was used to quantitate the CD4+Treg frequency and expression of Ki67, pSTAT5, FoxP3, CD25, and Helios. Data represent mean ± SEM from three independent animals.

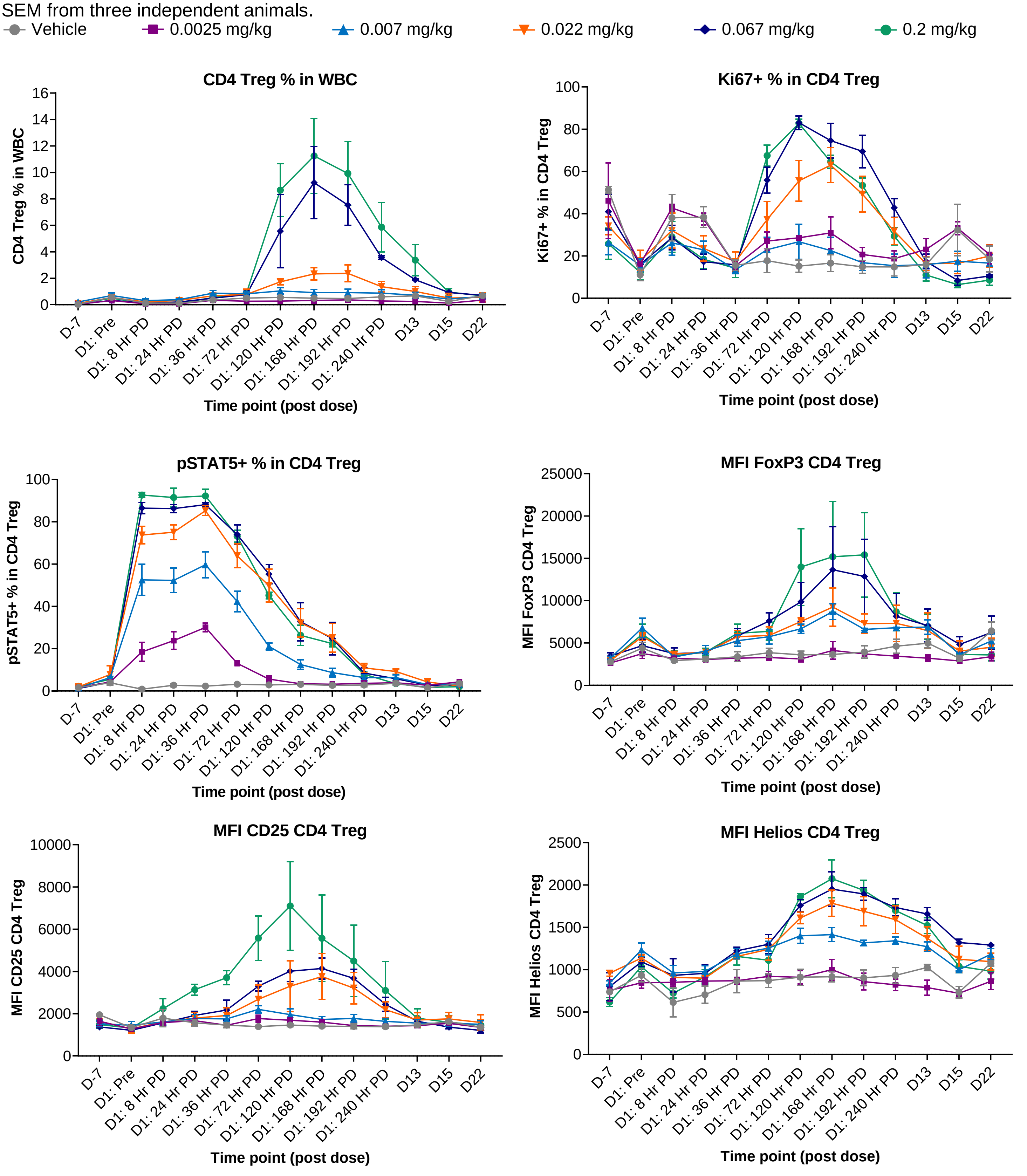

Supplementary Table 1. Antibodies used for flow cytometry.

Species	Antigen	Fluorophore	Clone	Vendor	Catalog #	Dilution
Human	CD3	A488	UCHT1	Biolegend	300415	1:10
	CD4	PE/Cy7	RPA-T4	Biolegend	300512	1:10
	CD8	PerCP/Cy5.5	RPA-T8	BD	560662	1:10
	CD8	SB780	RPA-T8	eBioscience	78-0088-42	1:50
	CD127	eF506	eBioRDR5	eBioscience	69-1278-42	1:10
	CD25	APC	M-A251	Biolegend	356110	1:10
	CD56	BV421	HCD56	Biolegend	318328	1:10
Mouse	Fc block	-	93	Biolegend	101320	1:10
	FoxP3	APC	FJK-16S	eBioscience	17-5773-82	1:10
	CD25	PE/Cy7	3C7	Biolegend	101916	1:10
	CD8	APC	53-6.7	Biolegend	100712	1:10
	CD4	PerCP/Cy5.5	GK1.5	Biolegend	100434	1:10
	CD127	PE	A7R34	Biolegend	135010	1:10
	CD4	APC/Cy7	L3T4	eBioscience	A15384	1:100
	CD45RB	APC	C363.16A	eBioscience	17-0455-81	1:100
	CD25	PE	PC61.5	eBioscience	12-0251-82	1:100
	CD3	PerCP/Cy5.5	145-2C11	BD	553067	1:10
	CD4	BUV737	RM4-5	BD	612844	1:10
	CD8	PE/CF594	53-6.7	BD	562283	1:10
	CD19	BV510	SJ25C1	BD	562947	1:10
	CD25	APC	PC61	BD	557192	1:10
	NK1.1	PE Cy7	PK136	BD	552878	1:10
	FoxP3	BV421	MF-23	BD	562996	1:10
	CD45	BV510	30-F11	BD	563891	1:20
	TCRb	BV711	H57-597	BD	563135	1:20
	CD45	FITC	RM4-5	BD	553047	1:20
	CD8a	PE	53-6.7	BD	553033	1:20
	CD25	BV786	3C7	BD	564368	1:10
	CD44	PE/Cy7	IM7	BD	560569	1:40
	CD62L	BV650	MEL-14	BD	564108	1:5
	FoxP3	V450	MF23	BD	561293	1:10
NHP	CD3	A700	SP34-2	BD	8162839	1:50
	CD4	BV711	OKT4	BioLegend	317440	1:20
	CD7	BV510	M-T701	BD	563650	1:20
	CD8	BUV805	SK1	BD	564912	1:20
	CD14	BV785	M5E2	BioLegend	301840	1:200
	CD16	BUV737	3G8	BD	564434	1:200
	CD20	BV785	2H7	BioLegend	302356	1:200
	CD25	Biotin	M-A251	BioLegend	356124	1:100
	FoxP3	PE	259D	BioLegend	320208	1:20
	ICOS	BV421	C398.4A	BioLegend	313524	1:100
	Helios	A488	D8W4X	CST	56424BC	1:25
	Streptavidin	BUV395	n/a	BD	564176	1:250
	Ki-67	PE/Cy7	SolA15	eBioscience	25-5698-82	1:500

Supplementary Table 2. Potency of IL-2 variants in CD8 T cells and Tregs.

Compound	NK cells (pg/mL)	CD8+ T cells (pg/mL)	Treg cells (pg/mL)	CD8+/Treg ratio
**rhIL-2**	4586	31024	75	414
**V91K[1]**	20537	102255	142	720
**S127AzK**	20210	92190	150	615
**T131AzK**	24207	132922	258	515
**T123AzK**	33396	152928	269	569
**E100AzK**	35088	195823	483	405
**N119AzK**	34010	143380	535	268
**M23AzK**	143620	539824	1030	524
**K9AzK**	169578	1100679	2217	496
**N26AzK**	258531	1188859	2459	483
**N88R[2]**	2312847	15025734	11082	1356
**Q126AzK**	3676807	19722480	29454	670
**H16AzK**	2545257	12070108	34976	345
**L19AzK**	6756768	22436430	93205	241
**N88R/D109AzK[3]**	2780819	12503386	175805	71
**N88AzK**	3298113	11111537	323201	34
**D20AzK**	2643930	9505217	1129455	8

[1]Compound based on Gavin MA. US Patent US20140286898A1. Interleukin-2 muteins for the expansion of t-regulatory cells. 2014.
[2]Shanafelt, A. B. et al. A T-cell-selective interleukin 2 mutein exhibits potent antitumor activity and is well tolerated in vivo. Nat. Biotechnol. 18, 1197–1202 (2000).
[3]Compound based on Greve J. US Patent US10035836B1. Modified IL-2 variants that selectively activate regulatory T cells. 2015. AzK was incorporated at D109 position and compound was pegylated using the pegylation strategy employed for other variants.

Supplementary Table 3. IL-2 and SAR'336 binding constants to human IL-2 receptor.

Sample	Surface	k_a ($M^{-1}s^{-1}$)	k_d (s^{-1})	K_D (nM)
**IL-2**	IL-2Ra	1.3e8	1.2	9.29
	IL-2Rb	9.00e5	0.2634	293
**SAR'336**	IL-2Ra	3.88e6	0.1257	32.4
	IL-2Rb	NO BINDING DETECTED		

Supplementary Table 4. EC50 (pg/mL) of IL-2 and SAR'336 by pSTAT5 detection in human cells.

Variant	Donor	*CD8 T cell*	*NK cell*	*Treg*
**IL-2**	1	12,669	6,047	24
	2	11,120	4,020	21
	3	14,590	3,010	19
	4	16,537	4,307	34
**SAR'336**	1	-	-	1,499,594
	2	-	-	2,223,949
	3	-	-	928,362
	4	-	-	1,824,464
