# Supplementary material for: A CD25-biased interleukin-2 for autoimmune therapy engineered via a semi-synthetic organism
Source: Commun Med (Lond). 2024 Mar 26;4:58. doi: 10.1038/s43856-024-00485-z (PMC10966033; doi:10.1038/s43856-024-00485-z)
Supplement: Supplementary file 2 — Description of Additional Supplementary Files [file 43856_2024_485_MOESM2_ESM.pdf]

Description of Additional Supplementary Files

File name: Supplementary Data 1
Description: Source data for the figures
