# Supplementary material for: A CD25-biased interleukin-2 for autoimmune therapy engineered via a semi-synthetic organism
Source: Commun Med (Lond). 2024 Mar 26;4:58. doi: 10.1038/s43856-024-00485-z (PMC10966033; doi:10.1038/s43856-024-00485-z)
Supplement: Supplementary file 4 — Reporting Summary [file 43856_2024_485_MOESM4_ESM.pdf]

nature research

Corresponding author(s): Marcos Milla
Last updated by author(s): Dec 1st, 2023

Reporting Summary

Nature Research wishes to improve the reproducibility of the work that we publish. This form provides structure for consistency and transparency in reporting. For further information on Nature Research policies, see our Editorial Policies and the Editorial Policy Checklist.

Statistics

For all statistical analyses, confirm that the following items are present in the figure legend, table legend, main text, or Methods section.

n/a	Confirmed	
☐	☒	The exact sample size (*n*) for each experimental group/condition, given as a discrete number and unit of measurement
☐	☒	A statement on whether measurements were taken from distinct samples or whether the same sample was measured repeatedly
☐	☒	The statistical test(s) used AND whether they are one- or two-sided<br>*Only common tests should be described solely by name; describe more complex techniques in the Methods section.*
☒	☐	A description of all covariates tested
☒	☐	A description of any assumptions or corrections, such as tests of normality and adjustment for multiple comparisons
☐	☒	A full description of the statistical parameters including central tendency (e.g. means) or other basic estimates (e.g. regression coefficient) AND variation (e.g. standard deviation) or associated estimates of uncertainty (e.g. confidence intervals)
☐	☒	For null hypothesis testing, the test statistic (e.g. *F*, *t*, *r*) with confidence intervals, effect sizes, degrees of freedom and *P* value noted<br>*Give P values as exact values whenever suitable.*
☒	☐	For Bayesian analysis, information on the choice of priors and Markov chain Monte Carlo settings
☒	☐	For hierarchical and complex designs, identification of the appropriate level for tests and full reporting of outcomes
☒	☐	Estimates of effect sizes (e.g. Cohen's *d*, Pearson's *r*), indicating how they were calculated

Our web collection on statistics for biologists contains articles on many of the points above.

Software and code

Policy information about availability of computer code

Data collection	Flow cytometry- BD LSR II or Fortessa, and ThermoFisher Attune NxT<br>Plate reader (Discoverx study)- PerkinElmer EnvisionTM<br>Biacore (SPR) software package
Data analysis	Structure analysis and alignment- Pymol<br>PK analysis- Phoenix WinNonlin 8.1<br>SPR analysis- Scrubber v2.0h<br>Flow cytometry analysis, fitting- Spotfire<br>Flow cytometry analysis, CellEngine (PrimityBio), and FlowJo v10<br>Plotting and statistical analysis- GraphPad Prism 8 XML<br>Plate reader (PK analysis)- SoftMax Pro (v7.1)

For manuscripts utilizing custom algorithms or software that are central to the research but not yet described in published literature, software must be made available to editors and reviewers. We strongly encourage code deposition in a community repository (e.g. GitHub). See the Nature Research guidelines for submitting code & software for further information.

Data

Policy information about availability of data

All manuscripts must include a data availability statement. This statement should provide the following information, where applicable:

- Accession codes, unique identifiers, or web links for publicly available datasets
- A list of figures that have associated raw data
- A description of any restrictions on data availability

The datasets generated and/or analysed during the studies reported herein are available from the corresponding author on reasonable request.

Field-specific reporting

Please select the one below that is the best fit for your research. If you are not sure, read the appropriate sections before making your selection.

☒ Life sciences ☐ Behavioural & social sciences ☐ Ecological, evolutionary & environmental sciences

For a reference copy of the document with all sections, see nature.com/documents/nr-reporting-summary-flat.pdf

Life sciences study design

All studies must disclose on these points even when the disclosure is negative.

Sample size	Sample sizes were not statistically pre-determined<br>Appropriate sample size were selected for each experiment to allow meaningful statistical confidence where required<br>For initial screening studies using the Discoverx assay, each compound was tested in duplicate to ensure reproducibility to identify potential hit compounds and discern from pharmacologically inappropriate variants. For pSTAT5 flow studies, sample size were selected to minimize natural donor variability effects and allow mean and SEM calculations for potency data. For in vivo studies, sample size was selected to balance animal use with statistical power, and allow temporal sampling density required to observed the effects in cases where terminal collections were required (PK/PD studies). For PK/PD studies, three animals per time point per compound were selected as appropriate to minimize animal to animal variability and statistical measure of the standard error of the mean effect. For tumor studies, sample sizes were selected to balance animal use and statistical power, and to minimize animal to animal variability effects on the results.
Data exclusions	In SPR study, rhIL-2 concentrations of 5-10uM produced aberrant signals that were not able to be fit. These signals saturated the surface and were excluded from the analysis to avoid artifacts in the fitting.
Replication	Biochemical and in vitro studies were performed with built-in replicates and multiple donors where applicable and described in methods section. In vivo studies (PK/PD studies, tumor efficacy) were confirmed in different models and will be reported elsewhere. No attempt to replicate any study was unsuccessful.
Randomization	Animals were allocated to study groups at random by the vendor or assigned associate and did not control for confounders such as order of treatments.
Blinding	All studies reported herein report quantitative data measured without subjective scoring, blinding was not relevant to these studies.

Reporting for specific materials, systems and methods

We require information from authors about some types of materials, experimental systems and methods used in many studies. Here, indicate whether each material, system or method listed is relevant to your study. If you are not sure if a list item applies to your research, read the appropriate section before selecting a response.

Materials & experimental systems

n/a	Involved in the study	
☐	☒	Antibodies
☒	☐	Eukaryotic cell lines
☒	☐	Palaeontology and archaeology
☐	☒	Animals and other organisms
☒	☐	Human research participants
☒	☐	Clinical data
☒	☐	Dual use research of concern

Methods

n/a	Involved in the study	
☒	☐	ChIP-seq
☐	☒	Flow cytometry
☒	☐	MRI-based neuroimaging

Antibodies

Antibodies used	All antibodies used in the studies reported are described in detail in the supplemental materials section of the manuscript.

Validation	All antibodies used for the studies reported are commercially available and have been validated by the manufacturer for the intended use.

Animals and other organisms

Policy information about studies involving animals; ARRIVE guidelines recommended for reporting animal research

Laboratory animals	C57BL/6 female mice 6-8 weeks of age with an average weight of 16 to 22 grams were purchased from Jackson Laboratories (Sacramento, CA) by Crown Biosciences. Purpose-bred cynomolgus monkeys (Macaca fascicularis) were sourced from licensed vendors and underwent standard quarantine periods prior to study initiation. Animal studies were conducted in AAALAC-accredited facilities at Charles River Laboratories (Reno, NV) under protocols approved by the Institutional Animal Care and Use Committee. 2- to 4-year-old male cynomolgus monkeys weighing 2-3 kg were used.
Wild animals	*Provide details on animals observed in or captured in the field; report species, sex and age where possible. Describe how animals were caught and transported and what happened to captive animals after the study (if killed, explain why and describe method; if released, say where and when) OR state that the study did not involve wild animals.*
Field-collected samples	*For laboratory work with field-collected samples, describe all relevant parameters such as housing, maintenance, temperature, photoperiod and end-of-experiment protocol OR state that the study did not involve samples collected from the field.*
Ethics oversight	In vivo studies were conducted by qualified personnel by Crown Biosciences and Charles River Laboratories and were performed in accordance with the approved IACUC protocol and Standard Operating Procedures.

Note that full information on the approval of the study protocol must also be provided in the manuscript.

Flow Cytometry

Plots

Confirm that:

- [x] The axis labels state the marker and fluorochrome used (e.g. CD4-FITC).
- [x] The axis scales are clearly visible. Include numbers along axes only for bottom left plot of group (a 'group' is an analysis of identical markers).
- [x] All plots are contour plots with outliers or pseudocolor plots.
- [x] A numerical value for number of cells or percentage (with statistics) is provided.

Methodology

Sample preparation	Sample preparation methods are described in detail in supplemental methods section
Instrument	Becton Dickinson Fortessa, LSRII, or ThermoFisher Attune NxT instruments
Software	FlowJo or CellEngine (PrimityBio)
Cell population abundance	Data available upon request
Gating strategy	Gating strategies described in detail in methods section, and is similar to what was used in Ptacin et.al 2021.

- [ ] Tick this box to confirm that a figure exemplifying the gating strategy is provided in the Supplementary Information.
